# Förster Resonance Energy Transfer (FRET) Demonstrates In Vitro Chitosan-Coated Nanocapsules Suitability for Intranasal Brain Delivery

**DOI:** 10.1021/acsami.5c01920

**Published:** 2025-04-28

**Authors:** Maria Alleva, Zsuzsa Baranyai, Natalia Esteban-Pérez, Pablo Martínez-Vicente, Rafael Martín-Rapún, María Moros, Jesús Martínez de la Fuente

**Affiliations:** † Instituto de Nanociencia y Materiales de Aragón (INMA), CSIC-Universidad de Zaragoza, Zaragoza 50009, Spain; ‡ Centro de Investigación Biomédica en Red de Bioingeniería, Biomateriales y Nanomedicina (CIBER-BBN), Madrid 28029, Spain; § Departamento de Bioquímica y Biología Molecular y Celular, Facultad de Ciencias de la Salud y el Deporte, Universidad de Zaragoza, Huesca 22002, Spain

**Keywords:** chitosan-coated nanoemulsions, nanocapsule integrity, Förster resonance energy transfer (FRET), nose-to-brain delivery, epithelial barrier model

## Abstract

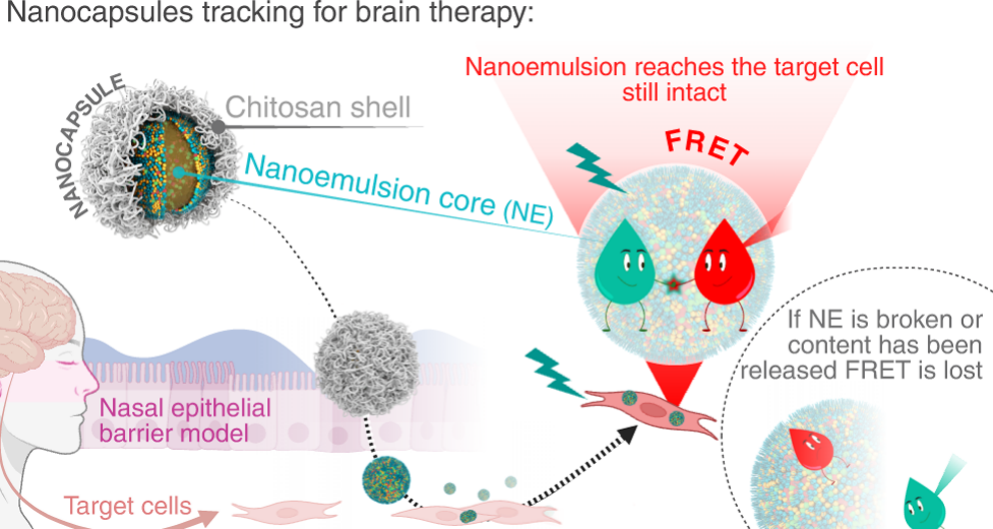

Intranasal drug delivery to the brain offers a promising strategy to overcome biological barriers. Chitosan-coated nanoemulsion-based nanocapsules demonstrate significant potential due to their mucoadhesive properties, ability to permeate epithelial cells, and ability to solubilize poorly water-soluble drugs, making them ideal candidates for bypassing the blood-brain barrier and overcoming the nasal mucosa. To ensure effective drug delivery, it is critical to assess the integrity of these nanocapsules during their transit across such barriers. In this study, we employed Förster resonance energy transfer to track the structural integrity of nanocapsules during transport. A simplified in vitro model was established using Calu-3 cells to mimic the mucosal epithelial barrier and Balb-c 3T3 fibroblasts as target cells. Our findings demonstrated that the nanoemulsion core of the nanocapsules successfully crossed the in vitro epithelial barrier and reached target cells while maintaining its structural integrity. These results validate the potential of chitosan-coated nanocapsules as a robust platform for the intranasal delivery of drugs to the brain.

## Introduction

The intranasal route holds significant potential for treating brain diseases by bypassing the blood-brain barrier.^1−4^ However, one major challenge in designing drug delivery systems is overcoming the mucus layer and epithelial cells, which could hamper drug delivery. These barriers can cause nanocarriers to degrade or disrupt, prematurely releasing their therapeutic payload. This complexity requires advanced tools to study the integrity and fate of these nanocarriers, allowing for a better understanding of how the payload is released and enabling continuous improvements in the customization of the carrier for tailored therapy.

Chitosan (CS), a natural cationic polysaccharide composed of β-(1 → 4)-linked d-glucosamine and *N*-acetyl-d-glucosamine, stands out due to its biodegradability, biocompatibility, mucoadhesion, and ability to facilitate drug transport across epithelia.^5,6^ Notably, CS can facilitate the transfer of macromolecules across epithelia, such as the nasal one.^7−9^ CS-based nanocarriers have been successfully applied in various drug delivery systems, including intranasal delivery.^5,10−14^ CS has mucoadhesive properties due to its interaction with negatively charged glycoproteins in the mucus, ensuring extended retention at the absorption site, reducing mucociliary clearance, and promoting sustained drug release while enhancing the overall bioavailability of the CS-coated nanocarrier.^1,15^ Furthermore, the ability to modify CS allows for fine-tuning the interactions between nanoparticles and mucus, optimizing their retention and diffusion into the mucus for enhanced epithelial uptake.^16^

In vivo studies have provided evidence of nose-to-brain delivery for CS-coated nanoemulsions (NEs), showing cargo transport via the trigeminal and olfactory nerves to the olfactory bulb, albeit in minute quantities.^9^ NEs are metastable colloids formed by dispersing one immiscible fluid within another and being stabilized by surfactants. Due to the small droplet size and high surface area of NEs, encapsulation of hydrophobic substances enhances their solubility and increases their bioavailability.^17−19^ In our group, we previously reported the preparation of CS-coated nanoemulsion-based nanocapsules (NCs) for drug delivery purposes.^20−22^ We used these NCs as an efficient intranasal drug delivery system for Alzheimer’s disease (AD), showing that NCs loaded with the p38 MAPK hydrophobic inhibitor could reach the target site—the cerebral cortex and hippocampus—after in vivo intranasal administration in the AD mice model. Inhibitor-loaded NCs outperformed the free drug, suggesting a direct transport route of the inhibitor from the nasal mucosa to the cortex.^23^ While NCs demonstrated their potential for brain-restricted reduction of p38 MAPK activity, the exact mechanisms underlying their transport as well as the role of nanocarrier integrity in enhancing therapeutic efficacy remain unclear. Understanding whether NCs maintain their structural integrity during transport is crucial for optimizing their therapeutic potential.

Advanced techniques such as magnetic resonance imaging (MRI) and positron emission tomography (PET) are commonly employed to track nanoparticles, utilizing magnetic properties and radioactive tracers to visualize their distribution.^24^ Similarly, fluorescence-based methods allow for real-time imaging within biological systems.^9^ However, these approaches often struggle to determine whether nanoparticles remain intact or whether their contents have been released, limiting their ability to assess nanocarrier stability after administration. Förster resonance energy transfer (FRET) stands out as the most effective method for studying nanocarrier integrity, enabling precise real-time monitoring of structural stability and payload release.^25−27^ FRET relies on the energy transfer between two fluorophores incorporated in the nanocarrier: a donor and an acceptor.^28^ The efficiency of this energy transfer depends on their proximity, typically within 2–10 nm, making FRET an ideal technique to monitor the structural integrity of nanoparticles.^29,30^ If the nanocarrier remains intact, the donor and acceptor will stay in close proximity, resulting in a detectable FRET signal. Conversely, if the nanocarrier disintegrates, the distance between the fluorophores increases, diminishing the FRET signal and indicating payload release.

**Scheme 1:**
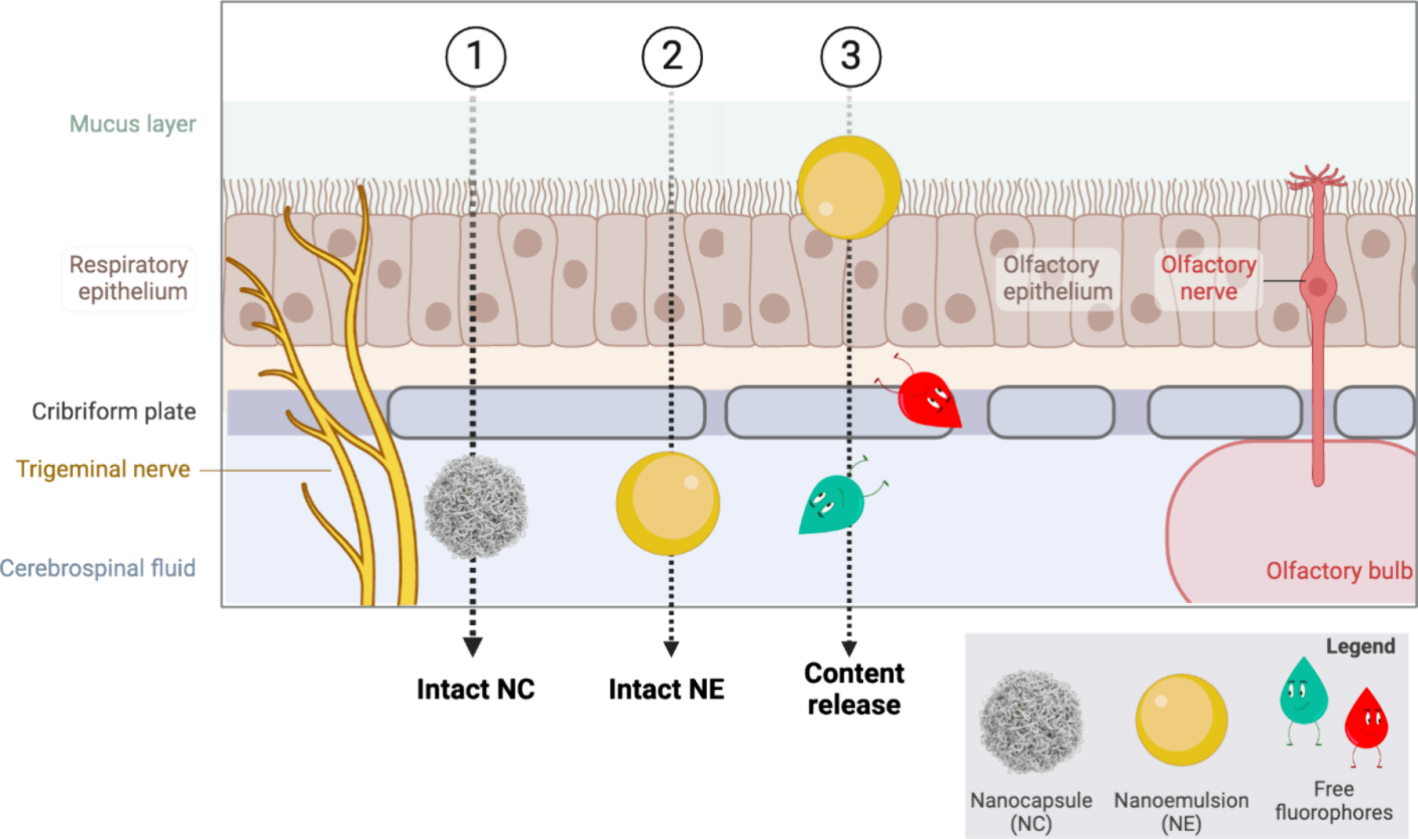
Main Hypotheses of the Fate of the NCs after Intranasal Administration (1) NCs are capable of crossing the nasal mucosal epithelium and reaching the brain still intact; (2) NCs are retained in the mucus and epithelial cells, where the CS shell disrupts and allows the NE to penetrate the mucosal epithelium; (3) NCs are retained in the mucus and epithelial cells and NEs release their content,which reaches the brain.

In this study, our goal was to better understand the fate of NCs, composed of an NE core coated with a CS shell, after administration to a simplified epithelial model (Scheme 1). Specifically, we explored whether: (1) the NCs could cross intact the nasal mucosal epithelium, potentially reaching the brain; (2) the CS shell was retained in the nasal mucus or the epithelial cells, while the inner NE could penetrate through the epithelium; and (3) the NCs could not cross the epithelial barrier but could still release their contents, which then reached the brain. To investigate which of these scenarios was taking place, we employed a FRET pair of lipophilic dyes^26,31^ to track the fate and integrity of the NCs when they cross an in vitro nasal epithelium barrier model. We conducted two approaches: (i) We loaded the NE core with the cyanine dye DiA (donor) and functionalized the CS shell with sulfo-Cyanine 5 (sCy5, acceptor). This approach would allow us to monitor the integrity of the NCs, specifically the CS shell and NE core through FRET, derived from the proximity of the fluorophores. (ii) We encapsulated DiA (donor) and another cyanine dye, DiD (acceptor), within the NE core of the NCs. In this case, generating an efficient FRET signal would enable us to track the integrity of the NE core and the cargo release.

## Experimental Section

### Materials

Tween 20, absolute ethanol (EtOH), NaH_2_PO_4_, and Na_2_HPO_4_ were purchased from Panreac Qumica S.L.U. (Barcelona, Spain). Span 85 (sorbitane trioleate), oleic acid, sodium tetraphenylborate (TPB), chitosan (ref: 448877, 75–85% deacetylated chitin, medium molecular weight, MW 190–310 kDa), ethyl acetate, dichloromethane (DCM), methanol (MeOH), D_2_O, TFA, Na_2_SO_4_, NaHCO_3_, Na_2_CO_3_, and 3-(trimethylsilyl)propionic-2,2,3,3-d_4_ acid sodium salt were purchased from Sigma-Aldrich Pte. Ltd. (Singapore). Water (double-processed tissue culture, endotoxin-free) used in all nanocapsule synthesis was from Sigma-Aldrich. The fluorophores DiA (4-Di-16-ASP (4-(4-(dihexadecylamino)styryl)-*N*-methylpyridinium iodide, Invitrogen D3883) and DiD (1,1′-dioctadecyl-3,3,3′,3′-tetramethylindodicarbocyanine perchlorate, Invitrogen D307) were purchased from Thermo Fisher Scientific (Madrid, Spain). Sulfo-Cyanine 5 NHS ester (sCy5-NHS ester) was obtained from Lumiprobe GmbH (Hannover, Germany). Phosphate buffered saline (PBS, 1X, pH 7.4, Gibco), Dulbecco’s phosphate buffered saline (DPBS, 1X, pH 7.4, Gibco), Minimum Essential Medium (MEM, Gibco), Dulbecco’s modified Eagle’s medium (DMEM, Gibco), 100 U/mL of penicillin/streptomycin, paraformaldehyde (PFA), 4′,6-diamidine-2′-phenylindole dihydrochloride (DAPI, Invitrogen D1306), hexamethyldisilazane (HMDS, 98%), and ZO-1 monoclonal antibody conjugated with Alexa Fluor 488 were from Thermo Fisher Scientific. Bovine serum albumin (BSA), fetal bovine serum (FBS), glutaMAX, sodium pyruvate, trypsin-EDTA (0.25% porcine trypsin and 0.2% EDTA), sodium cacodylate trihydrate, osmium tetroxide (OsO4), uranyl acetate, lead citrate, xylene, alcian blue (AB), and Triton X-100 were purchased from Sigma-Aldrich. Glutaraldehyde was purchased from Electron Microscopy Sciences (Hatfield, PA, USA). Carbon-coated 200-mesh copper grids were supplied by Agar Scientific Supplies.

### Methods

#### CS Functionalization with sCy5-NHS Ester

For the synthesis of sCy5-labeled CS, different substitution degrees were planned: 0.2, 1, and 5%. One mg of CS has 4.7 μmol amino groups as calculated from the mean deacetylation degree (80%) of the applied CS. This ratio was used for calculating the theoretical substitution degrees of the functionalized CS. First, 12 mg of CS (with 56.4 μmol amino group theoretically, 2.4 mL of 5 mg/mL CS solution in acetic acid 1% (v/v)) was added to 10 mL of 0.1 M NaHCO_3_/Na_2_CO_3_ buffer (pH 8.5) in each case. sCy5-NHS ester (MW: 777.95 Da, 112.8 nmol for 0.2%, 564 nmol for 1%, and 2.82 μmol for 5% theoretical substitution degree, from 10 mg/mL sCy5-NHS ester stock solution in DMSO) was added to the CS solution, the reaction was left under magnetic stirring during 8 h (300 rpm, RT), and an amide bond was formed. Finally, the reaction solutions of the different functionalized CSs were transferred into dialysis bags (SnakeSkin dialysis tubing, 3.5 K MWCO, Thermo Fisher Scientific, Waltham, MA, USA) and subjected to dialysis against 2 L water for 72 h. The water was changed every 24 h during the dialysis process. Then, the CS solutions were freeze-dried to obtain the solid product. For NC synthesis, sCy5@chitosans were dissolved to obtain 5 mg/mL concentration in water with 1% acetic acid (v/v). For fluorescence measurements, the sCy5@CSs solutions were diluted with MeOH to obtain 5 μg/mL solution and their fluorescence intensity was measured with excitation at 600 nm and detection at 662 nm using a BioTek Synergy H1 microplate reader (Agilent Technologies, Santa Clara, California, USA) after carrying out a calibration curve of sCy5-NHS ester in MeOH (Figure S1) and used to calculate the measured substitution degree of sCy5@CSs (Table S1).

#### Counterion Substitution of DiA and DiD Fluorophores

DiA-TPB and DiD-TPB were synthesized based on previously described methods (Figure S2).^26,32^ Briefly, for DiA-TPB, 6.2 mg (7.8 μmol) of DiA-iodide was mixed with 20 mol equiv of sodium TPB (53.6 mg, 156 μmol) in 1.5 mL of ethyl acetate/DCM 2:1 (v/v). For DiD-TPB, 5.5 mg (5.7 μmol) of DiD-perchlorate was mixed with 20 mol equiv of sodium TPB (39.2 mg, 114 μmol) in 1 mL of ethyl acetate, where both salts readily dissolved. After 1 h of magnetic stirring, the formation of the TPB salts was confirmed by thin-layer chromatography (TLC aluminum sheets, silica gel layer, ALUGRAM Xtra SIL G UV254, MACHEREY-NAGEL GmbH, Düren, Germany) using DCM/MeOH, 95:5 (v/v), where the TPB products exhibited a retardation factor significantly higher than that of the initial compounds. The solvent was evaporated by a rotary evaporator, and the TPB salts were purified by column chromatography (DCM/MeOH, 95:5 (v/v), silica gel (high-purity grade, average pore size 60 Å, Sigma-Aldrich)). The desired fractions were pooled, the solvent was evaporated, the resulting TPB salts were weighed, and stock solutions of DiA-TPB and DiD-TPB in EtOH were prepared.

### Preparation of the Fluorophore-Loaded NCs

#### Synthesis of DiA-Loaded sCy5@CS-NCs

The synthesis of the NCs was carried out as previously described^20,21^ with modifications. To encapsulate DiA-TPB, 0.2 μmol (461 μL of 434 μM DiA-TPB in EtOH) was added to 4 mL of absolute ethanol containing 40 mg of oleic acid and 8.6 mg of Span 85, obtaining the organic phase.

For the formation of the NE core, this organic phase was added drop by drop under magnetic stirring to the aqueous phase, which contained 13.6 mg of Tween 20 dissolved in 8 mL of water and left stirring for 15 min. Next, 750 μL of a 5 mg/mL sCy5@CS solution in 1% (v/v) acetic acid was added, allowing the formation of a CS coating on the NE core. Then, the mixtures were stirred for 15 min. To obtain the final polymeric hydrogel shell, the sCy5@CSs-coated NE was added to 15 mL of 50 mM Na_2_SO_4_ solution in water while gently stirring. The excess of Na_2_SO_4_ was removed by ultracentrifugation (20,000 g, 25 min, 10 °C), and NCs were resuspended in 2 mL of water. The concentration of the NCs in the water suspension was determined by measuring the weight of a sample after freeze-drying. The NCs were diluted to a concentration of 10 mg/mL and were freeze-dried with 10% (m/m) mannitol as cryoprotectant and were stored at 4 °C. The steps for the preparation of empty sCy5@CS-NCs followed the protocol presented above, adding the appropriate sCy5@CS.

#### Synthesis of DiA and DiD-Loaded NCs

To encapsulate fluorescent dyes in NCs, 0.2 μmol of DiA-TPB (461 μL of 434 μM DiA-TPB in EtOH) or 0.2 μmol of DiD-TPB (222 μL of 903 μM DiA-TPB in EtOH)—for single-dye-loaded NCs—or both DiA-TPB and DiD-TPB (0.2 μmol each)—for FRET-pair-loaded NC—were mixed with 4 mL of absolute ethanol containing 40 mg of oleic acid and 8.6 mg of Span 85, obtaining the organic phase. Fluorophores with the original counterion were also used for the preparation of the NCs. For this, 0.2 μmol of DiA-I (20 μL of 10 mM DiA-I in EtOH), or 0.2 μmol of DiD-PC (20 μL of 10 mM DiD-PC in EtOH), or both DiA-I and DiD-PC (0.2 μmol each) were added to the organic phase. For the formation of the NE core and the CS coating and to obtain the final polymeric hydrogel shell, we followed the protocol presented above. The concentration of the NCs in the water suspension was determined by measuring the weight of a sample after freeze-drying. Empty NCs as controls, without adding fluorophores, were also prepared using the same method. The obtained NCs were freeze-dried with mannitol, as described above.

### Encapsulation Efficiency and Fluorophore Loading

Encapsulation efficiency is the percentage of encapsulated fluorophores over the amount initially added to the preparation of nanocarriers. Fluorophore loading is the amount of fluorophore encapsulated (in nmol) per milligram (mg) of NC. To calculate the amount of encapsulated fluorophores in the NCs, the appropriate volume of suspension that contains 0.5 mg of NC was mixed with MeOH to obtain the final volume of 500 μL and sonicated for 30 min to achieve the complete extraction of the encapsulated dyes. This solution was then centrifuged (13,000 rpm, 5 min) to eliminate the CS fragments eventually present. The fluorescence was measured with excitation at 455 nm for DiA and 650 nm for DiD and detection at 586 nm for DiA and 670 nm for DiD using a BioTek Synergy H1 microplate reader after a calibration curve of each dye was carried out in MeOH (Figure S3).

### FRET Signal Evaluation by Fluorimetry and Flow Cytometry

Fluorescence emission spectra were recorded with a PerkinElmer LS 55 fluorescence spectrometer (PerkinElmer Inc., Shelton, CT, US) using quartz cells with a path length of 1 cm. The samples containing the fluorophores (0.1 μM for free fluorophores in EtOH and 0.3 mg/mL for NCs in water) were irradiated by using excitation wavelengths at 450, 488, 585, and 633 nm. The fluorescence emission spectra were recorded at room temperature with an excitation and emission slit width of 10 nm and an integration time of 1200 s. Emission spectra were collected from 450 to 700 nm with a 1 nm increment. The semiquantitative parameter of FRET efficiency (also called the proximity ratio (PR)) was calculated according to the following equation:
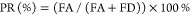
where FA and FD are, respectively, the acceptor and donor maximal emission intensities at the donor excitation wavelength. For the characterization, the selected emission wavelength for encapsulated DiA was 561 nm when excited at 450 nm. For encapsulated DiD, the selected emission wavelength was 671 nm when excited at 633 nm to avoid spectral overlap with the excitation source.

The fluorescent signal of the NCs in the cMEM was also characterized by a CytoFLEX type flow cytometer (Beckman Coulter Life Sciences, Indianapolis, IN, USA) using a 488 nm laser. The PE channel was used to detect DiA fluorescence (emission at 585/42 nm), and the PC5.5 channel to detect DiD and Cy5 fluorescence (emission at 690/50 nm). Data were analyzed by using CytExpert 2.4 software (Beckman Coulter) and Kaluza 2.1 software (Beckman Coulter).

To study FRET reduction or loss due to the disruption of the FRET-NCs, the NCs were added to complete MEM without phenol red at a concentration of 0.6 mg/mL NCs in 3 mL of medium. The fluorescence was checked before and after 5 min of sonication with a sonicator tip (UP400 St Ultrasonic Homogenizer, Hielscher Ultrasonics, Teltow, Germany) at 340 W of power.

### Zeta Potential and Size of the NCs

Dynamic light scattering (DLS) analysis and surface potential (zeta (ζ) potential) measurements were carried out using a Malvern Zetasizer Nano ZS (Malvern Panalytical Ltd., Malvern, United Kingdom). NC hydrodynamic diameter, polydispersity index (PDI), and ζ potential were measured in water at a concentration of 0.1 mg/mL at 25 °C.

### Transmission Electron Microscopy (TEM) of the NCs

TEM analysis was carried out using a Tecnai T20 microscope (Thermo Fisher Scientific) at 200 kV. The sample was first fixed in a centrifuge tube using 0.25% glutaraldehyde in 0.01 M phosphate buffer (based on NaH_2_PO_4_ and Na_2_HPO_4_) (v/v) for 1 h at 4 °C, then centrifuged and washed three times, each time in 0.01 M phosphate buffer for 1 min. A 10 μL drop of each sample was placed on Parafilm in a Petri dish. Freshly glow-discharged (30 s, 15 mA) carbon-coated 200-mesh copper grids were incubated for 5 min on the sample drops and then washed with a droplet of distilled water for 1 min. The grids were then stained with 2% uranyl acetate in water for 1 min, and the excess stain was removed by touching the edge of the grid with filter paper.

### NCs Stability and NE Diffusion through the CS Shell in Release Assays

Fluorescent NCs were introduced into PBS, chosen as the release medium, at a concentration of 1 mg/mL NCs at a final volume of 0.5 mL and left at 37 °C for 24 h in 2 mL centrifugal tubes under constant mechanical stirring (150 rpm). The release experiment was performed in duplicate. A schematic representation is provided in Scheme S1. At various time points, samples were filtered (Millex-GV Filter, 0.22 μm; PVDF, 13 mm; Merck Millipore, Darmstadt, Germany). The intact NCs retained in the filters were extracted by passing 500 μL of MeOH through the filter twice, which induces the degradation of the NCs, allowing the encapsulated molecules to dissolve in the solvent for subsequent quantification. The remaining NCs adhered to the walls of the centrifugal tubes were also extracted with 500 μL of MeOH. The amount of extracted fluorophores in MeOH that corresponds to the number of fluorophores still present in the NCs was determined by a BioTek Synergy H1 microplate reader by fluorescence measurements using calibration curves, excitation, and detection wavelengths presented above. The sum of filter and centrifugal tube extractions was considered for the complete release profile. Data presented were calculated as percentages, where 100% corresponds to the value representing the fluorophores retained in the NCs under storage conditions.

### Cell Culture Conditions

The Calu-3 human lung epithelial cell line (American Type Culture Collection, ATCC HTB-55, Manassas, VA, USA) was maintained in MEM supplemented with 10% FBS, 2 mM GlutaMAX, 1 mM sodium pyruvate, and 100 U/mL penicillin/streptomycin (complete MEM, cMEM). Balb-c 3T3 clone A31 mouse embryo fibroblast (American Type Culture Collection, ATCC CCL-163) was maintained in DMEM supplemented with 10% FBS, 2 mM GlutaMAX, 1 mM sodium pyruvate, and 100 U/mL penicillin-streptomycin (complete DMEM, cDMEM). Both cell types were cultured at 37 °C in with 5% CO_2_ and used for the experiments from passages 2 to 20 for Calu-3 and 10 to 13 for Balb-c 3T3. Cells were confirmed to be free of mycoplasma contamination.

### NC Transport across the Calu-3 Barrier Model

Calu-3 lung epithelial cells were seeded to Transwell (TW) inserts (polyethylene terephthalate, 3.0 μm pore size, 1.12 cm^2^ growth area) in Transwell cell culture chambers (Product Number 3462, Corning Costar, Cambridge, MA, USA) at a density of 400,000 cells/500 μL/insert (equal to 357,000 cells/cm^2^) and cultured for 10 days. Every 2 days, the culture medium from the apical and basal chambers was removed, cells were washed twice with 1 mL PBS, both chambers were filled with 1 mL PBS, and transepithelial electrical resistance (TEER) was measured to monitor monolayer formation on the inserts using a Millicell-ER-2 system (Millipore Corporation, Billerica, MA). Then, PBS was replaced with fresh cMEM (0.5 mL per insert (apical part) and 1 mL per well (basal part)) for further culturing. After 10 days in culture, cell monolayers with TEER values over 500 Ω·cm^2^ were used for experiments. Balb-c 3T3 fibroblasts were seeded into 12-well plates (TPP, Techno Plastic Products AG, Switzerland) at a density of 40,000 cells/600 μL/well 2 days before the permeability experiment. On the day of the experiment, both cell types were washed twice with PBS, and then TW inserts with Calu-3 monolayer (apical chamber) were placed on the 12-well plates containing the Balb-c 3T3 cells (basal chamber). The basal chambers were filled with 400 μL cMEM. NCs (DiA-NCs, DiD-NCs, FRET-NCs, and a mixture of DiA-NCs and DiD-NCs) at a concentration of 0.3 mg/mL in a volume of 200 μL were added to the apical chamber. Cells without NCs were employed as negative controls, and free fluorophores (DiA- and DiD-TPB) were also added at the same concentration as the encapsulated fluorophores. After 24 h, Balb-c 3T3 cells were washed with PBS, treated with 150 μL of trypsin-EDTA, and after 5 min of incubation at 37 °C, trypsin was inactivated by 750 μL cMEM. Then, cells were transferred from the plate to tubes, centrifuged (1500 rpm, 5 min), and the supernatant was removed. Cells were resuspended in 150 μL of 10% FBS containing PBS, and their intracellular fluorescence intensity was measured with a CytoFLEX flow cytometer using the same excitation and detection setting as described above. Data was analyzed as described above, for the gating strategy, see Figure S4. The transcellular transport across the Calu-3 cell monolayer was then assessed in triplicate to consider the biological variability from one well to another.

### Statistical Analysis

Data were analyzed using GraphPad Prism 8.0 (GraphPad Software Inc., Boston, MA, USA). The results are presented as the mean ± standard deviation from at least two independent experiments. One-way analysis of variance (ANOVA) followed by the Dunnet multiple comparison test was used to evaluate differences between groups. Data points were considered statistically significant at a *p* value of <0.05.

## Results and Discussion

### Preparation of the Fluorophore-Loaded NCs

**Figure 1:**
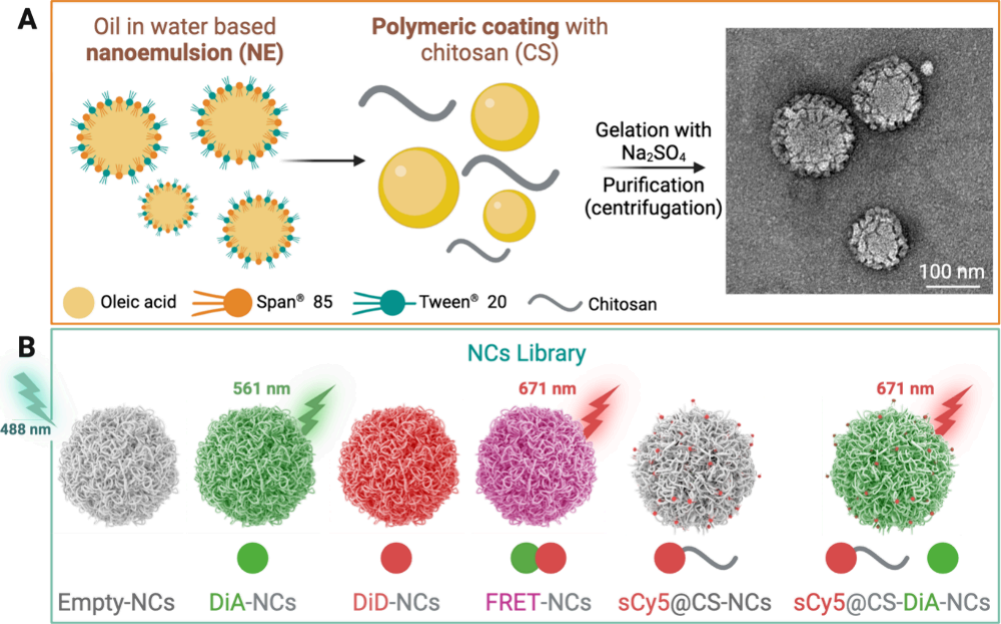
(A) Schematic representation of the NCs synthesis process and TEM micrograph of NCs. (B) Library of the synthesized NCs and graphical representation of the expected emission wavelength when excited at 488 nm (wavelength used in the following experiments to excite DiA in a flow cytometer).

The NCs are made of a lipophilic NE-based core composed of oleic acid and stabilized with Span 85 and Tween 20 surfactants that is further coated with a hydrophilic cationic CS hydrogel shell (Figure 1A).^20,21^ Our goal was to track both the overall integrity of the NCs and the NE core, providing insight into the eventual release of the NE from the CS shell as well as the potential release of the payload from the NE core. To achieve this, we developed two types of NCs: (i) one designed to monitor the entire NC structure by labeling the CS shell and the NE core and (ii) another focused on tracking the NE core itself.

(i) To track the two main components of the NCs—the CS outer shell and the NE inner core—we labeled CS with sCy5-NHS ester with three substitution degrees: 0.2, 1, and 5% (Table S1). Labeled CSs were characterized through NMR and FTIR analysis (Figure S5) and used to synthesize fluorescent NCs (empty and DiA-loaded). The overlapping emission and excitation spectra of the fluorophores (Figure S6) ensured the conditions necessary for efficient FRET detection in case of close proximity (distance less than 10 nm).^28^ NC colloidal stability and labeled CS fluorescent signal were used as a readout. Since NEs coated with CS with a 5% substitution degree were unstable and CS with a 0.2% substitution degree resulted in low fluorescence intensity (data not shown), only the 1% substitution degree was employed for further studies.

(ii) To specifically monitor the NE core and potential payload release, we prepared NCs with the FRET pair encapsulated within the NE core. For this purpose, we loaded the core with two cyanine dyes, DiA (donor) and DiD (acceptor), forming a FRET pair with compatible overlapping emission and excitation spectra (Figure S7).

For control purposes, additional NCs containing only DiA and DiD were synthesized. Furthermore, to enhance dye solubility in oil, ensure loading stability, and reduce dye leakage, the hydrophilic counterions of DiA and DiD—iodide (I) and perchlorate (PC), respectively—were replaced with the hydrophobic TPB counterion before encapsulation (Figure S2).^26,32^Figure 1B shows the library of NCs used in this study. From this point forward, we refer to the first type of NCs, where the CS shell is labeled with sulfo-Cyanine 5 (sCy5) and the NE core with DiA, as sCy5@CS-DiA-NCs. The second type, with the FRET pair DiA and DiD encapsulated within the NE core, will be referred to as FRET-NCs.

### Encapsulation Efficiency and Fluorophore Loading

The encapsulation efficiency (EE)—the amount of fluorophore encapsulated per amount of fluorophore initially added—and the fluorophore loading (FL)—the amount of fluorophore encapsulated (in nmol) per mg of NC—were determined as detailed in Material and Methods. As mentioned, we substituted the counterions of the fluorophores with a more lipophilic one (TPB) to minimize fluorophore leakage. We compared the results obtained encapsulating both fluorophores—with commercial or substituted counterions—in Table S2. In the case of FRET-NCs, switching from the original counterions to fluorophores-TPB resulted in an improvement in EE, while FL remained similar.

EE and FL values obtained for NCs containing DiA(TPB) or DiD(TPB) are shown in Table 1. DiA(TPB)-NCs and DiD(TPB)-NCs showed similar FL, while the EE was higher for DiA-TPB. FRET(TPB)-NCs showed a slightly lower EE value for DiA-TPB and a similar one for DiD-TPB compared to the NCs control. For FL, DiA-TPB content was similar, while DiD-TPB content slightly decreased.

**Table 1:** EE and FL Data of the NCs

NCs	EE, %	FL, nmol fluorophore/mg NC
DiA(TPB)-NCs	93.6 ± 6.4	4.5 ± 0.3
DiD(TPB)-NCs	54.6 ± 1.6	4.4 ± 0.1
FRET(TPB)-NCs	DiA: 76.6 ± 4.7	DiA: 4.2 ± 0.3
	DiD: 59.8 ± 6.0	DiD: 3.2 ± 0.3
sCy5@CS-DiA(TPB)-NCs	47.4 ± 0.4	2.4 ± 0.0

**Figure 2:**
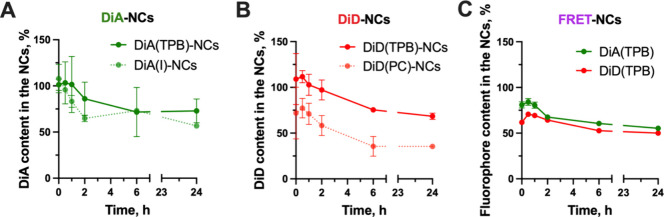
Fluorophore content inside the (A) DiA-NCs, (B) DiD-NCs, and (C) FRET-NCs during the release assay performed in PBS at 37 °C with a 1 mg/mL NC concentration.

We then performed the release assay by adding fluorescent NCs to PBS and keeping them under constant stirring at 37 °C for 24 h (Scheme S1).^21,23^ Samples were collected at different time intervals, and the amount of fluorophore remaining in the NCs was quantified using fluorimetry (Figure 2). Release profiles for DiA-loaded NCs showed no significant differences between iodide and TPB counterions (Figure 2A). Conversely, NCs loaded with the fluorophore DiD-TPB had a 25% higher retention of the fluorophore within the NE compared with NCs synthesized with the original counterion, DiD-PC (Figure 2B). These results are consistent with the findings of Roger et al.^26^ In any case, we selected TPB instead of I or PC since it helps mitigate the self-quenched π-stacked structures (H-aggregates) that cyanine dyes can form at high concentrations.^33^ Of note, after 24 h in PBS at 37 °C, the DiA and DiD content in all NCs with TPB counterion (DiD-, DiA-, and FRET-NCs) was around 70–80%, showing similar stability of encapsulation (Figure 2C). For these reasons, we opted to use DiD-TPB and DiA-TPB for further studies. Starting from here, for simplification reasons, DiA(TPB)-NCs, DID(TPB)-NCs, FRET(TPB)-NCs, and sCy5@CS-DiA(TPB)-NCs will be referred to as DiA-NCs, DiD-NCs, FRET-NCs, and sCy5@CS-DiA-NCs, respectively.

### Zeta Potential, Size, and Morphology of the NCs

From a biological point of view, an overall positive charge can significantly enhance cellular uptake, transport, and distribution of nanocarriers compared to negatively charged counterparts.^34,35^ This is primarily due to the electrostatic interactions between the positively charged nanocarriers and negatively charged cell membranes. We assessed the surface charge of all of the NCs by measuring their ζ potential (Table S3). Unmodified NCs showed a positive ζ potential ranging from +30 to +34 mV, indicating a stable colloidal suspension and that the NE was correctly coated with CS.^20^ In the case of NCs with the sCy5@CS coating, a lower ζ potential (+13 mV) was obtained because of the lower number of free amino groups in the CS. The lower ζ potential of sCy5@NCs may lead to reduced colloidal stability compared to the unmodified NCs. For intranasal delivery, positively charged particles are typically attracted to the mucus under normal conditions, as it displays negatively charged carboxylate and sulfate groups. This characteristic makes the CS-coated NCs well-suited for retention in the mucus layer.^36^ Enhanced retention in intranasal drug delivery improves absorption and bioavailability by prolonging the formulation residence time at the nasal mucosa and facilitating epithelial transport. It also reduces drug clearance by mucociliary action, enabling sustained release.^14,37,38^

The size of NCs also influences uptake efficiency as it affects their adhesion and interaction with cells. The hydrodynamic size of the NCs, measured by DLS, showed a size range between 75 and 202 nm (Table S3). This size range is adequate for obtaining high nasal mucosa permeability.^9^ Additionally, we performed electron microscopy characterization using both SEM (scanning electron microscopy, Figure S8A,B) and TEM (Figures 1A and S8C,D). Size distribution analysis was consistent with DLS, and NCs showed a spherical shape with an amorphous surface.

### FRET Signal Evaluation by Fluorimetry and Flow Cytometry

**Figure 3:**
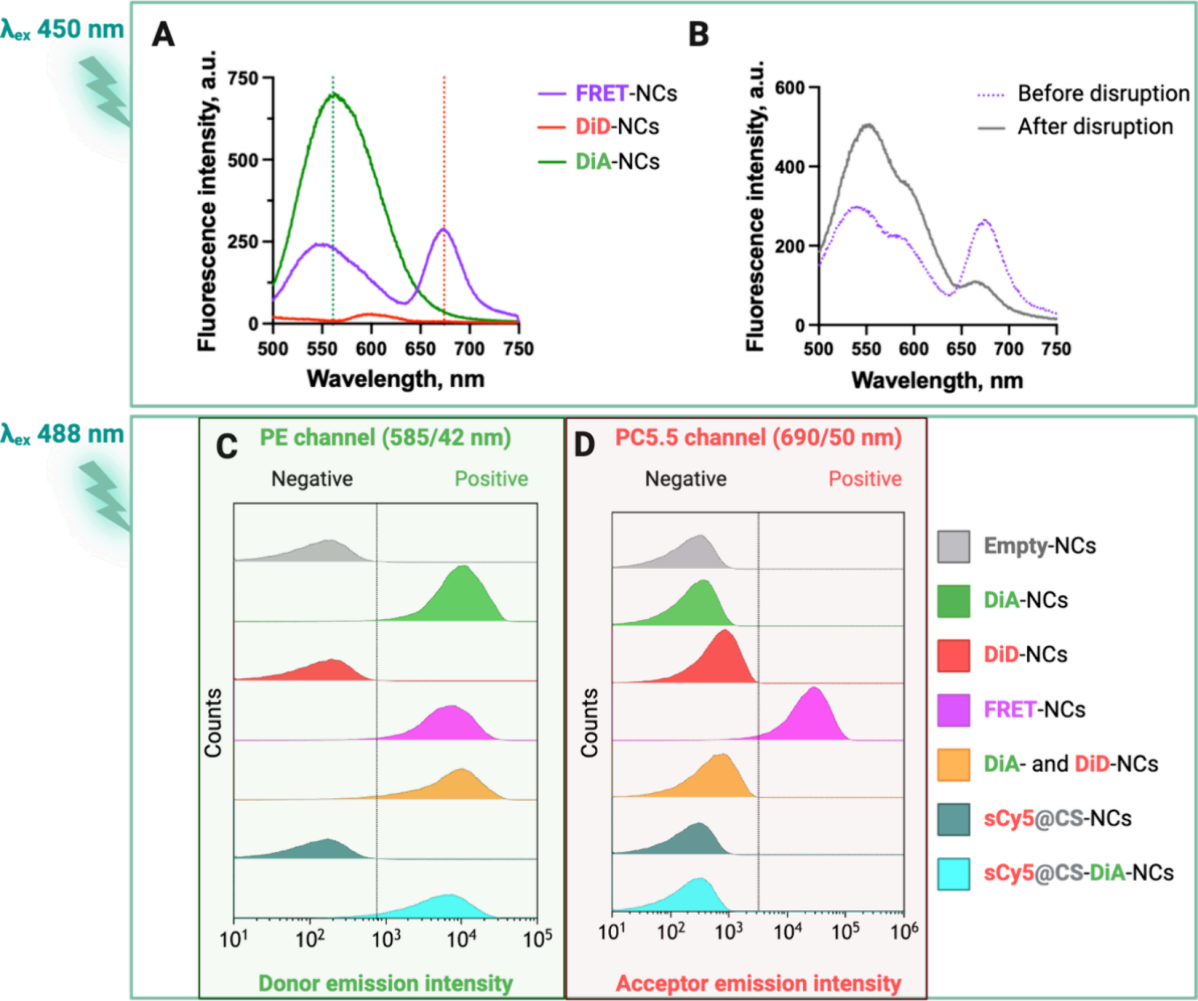
(A) Emission spectra of DiA-NCs, DiD-NCs, and FRET-NCs in water with an NC concentration at 0.3 mg/mL excited at λ_ex_ 450 nm. (B) FRET-NCs in cMEM with an NC concentration at 0.6 mg/mL before and after sonication. Flow cytometry analysis of NCs, the fluorescent signal of the NCs excited with a 488 nm laser and detected at (C) the PE channel (donor emission intensity, 585/42 nm) and at (D) the PC5.5 channel (acceptor emission intensity, 690/50 nm).

To evaluate the FRET phenomenon in the NCs, we employed fluorimetry (Figure 3A,B) and flow cytometry (Figure 3C,D). We first confirmed the presence of sCy5 in sCy5@CS-NCs and sCy5@CS-DiA-NCs by fluorimetry (Figure S9A). Then, to assess whether a FRET signal was present in sCy5@CS-DiA-NCs, we collected the fluorescence emission spectra at 671 nm with 450 nm excitation, corresponding to the donor (DiA) excitation wavelength (Figure S9B). No FRET signal was observed, indicating that the core and shell compartments are well-defined and that the distance between sCy5 in the shell and DiA in the core is greater than 10 nm. This separation is likely influenced by the surfactants used in the synthesis for NE stabilization that may create a layer between the core and shell.

For the FRET-NCs (double-dye loaded), we detected two emission peaks when excited at 450 nm: one at 561 nm, corresponding to the fluorescence of the donor, and another at 671 nm, indicating acceptor fluorescence and confirming that energy transfer was occurring (Figure 3A). The calculated PR value for FRET was approximately 50–60%, depending on the batches. Fluorescence intensity demonstrated a linear relationship with NC concentration with a proportional increase in fluorescence signals. At the same time, the PR remained constant across all concentrations, indicating stable emission properties of the NCs across varying concentrations (Figure S10). In the emission spectra of the control single-dye-loaded NCs excited at 450 nm, we observed a fluorescence signal at 561 nm for DiA-NCs, while no peak was noted for DiD-NCs as expected (Figure 3A). To further prove that FRET is a consequence of the coencapsulation, we intentionally disrupted the NCs, sonicating them, and then we checked their fluorescence again. The emission spectrum of the FRET-NCs before sonication (Figure 3B) was characterized by the presence of two main peaks. As expected, after sonication, the spectrum of the sample changed. Acceptor fluorescence intensity decreased, while donor fluorescence intensity was higher. The loss of the FRET signal and the recovery of donor fluorescence confirmed that when the NCs are disrupted, the spatial separation of the fluorophores hinders the energy transfer.^39^

To complement the results, we used flow cytometry for the FRET detection in NCs. The flow cytometry gating strategy of a representative sample of empty NCs is shown in Figure S11. We compared the fluorescence intensity of empty-NCs, DiA-NCs, DiD-NCs, FRET-NCs, sCy5@CS-NCs, and sCy5@CS-DiA-NCs by exciting the NCs with a 488 nm laser and detecting their signals in the PE channel (donor emission) and PC5.5 channel (acceptor emission) (Figure 3C,D). Additionally, to exclude potential fluorophore diffusion between particles, we analyzed a mixture of DiA- and DiD-NCs loaded separately. As expected from the results of fluorimetry, flow cytometry also confirmed the lack of FRET in the case of sCy5@DiA-NCs. The other DiA-containing systems (DiA-NCs, FRET-NCs, and the mixture of DiA- and DiD-NCs) showed fluorescence in the donor emission channel, while only FRET-NCs showed notable fluorescence in the acceptor emission channel. The lack of FRET in the control mixture (DiA- and DiD-NCs) confirmed that only when fluorophores are closely entrapped inside the NCs is their spatial distance enough to allow the occurrence of energy transfer. Notably, DiD-NCs excited at 488 nm exhibited a very low emission intensity at the PC5.5 channel, contributing minimally to the overall fluorescence intensity observed in the case of FRET-NCs.

Based on the absence of FRET in sCy5@CS-DiA-NCs, we determined that these NCs were not suitable for assessing whether NCs could cross the nasal mucosal epithelium intact and reach the brain, leading to their exclusion from further testing. However, the FRET signal provided by the NE of FRET-NCs, assessed via fluorimetry and flow cytometry, allows tracking the integrity and the penetration of the NE core through the mucosal epithelium after administration.

To evaluate FRET-NC stability, we analyzed the PR and hydrodynamic diameter in water, PBS, and cMEM over 24 h at 37 °C (Figure S12). At time 0, the PR values were approximately 50% in water, 37% in PBS, and 47% in cMEM. The observed differences in PR values between water and PBS can be attributed to solvent-dependent changes in electrostatic interactions and fluorophore environment, as reported in previous studies.^40,41^ After 24 h, the PR remained consistent in water and PBS, but it decreased by about 10% in cMEM, suggesting some NC disruption. The hydrodynamic diameter remained unchanged in water, but in PBS and cMEM, a smaller population appeared after 24 h. The observed decrease in hydrodynamic size in PBS and cMEM suggests possible structural rearrangements, which could be due to the loss of the CS shell. The high ionic strength in PBS can weaken the interactions between the CS shell and the NE core, potentially leading to shell detachment.^42^ Similarly, serum proteins in cMEM can adsorb onto the NCs surface, forming a protein corona that may displace the CS shell, leading to the loss of it.^43^ Future studies are necessary to elucidate these interactions and their impacts on NC stability and function.

### NCs Stability and NE Diffusion through the CS Shell in Release Assays

**Figure 4:**
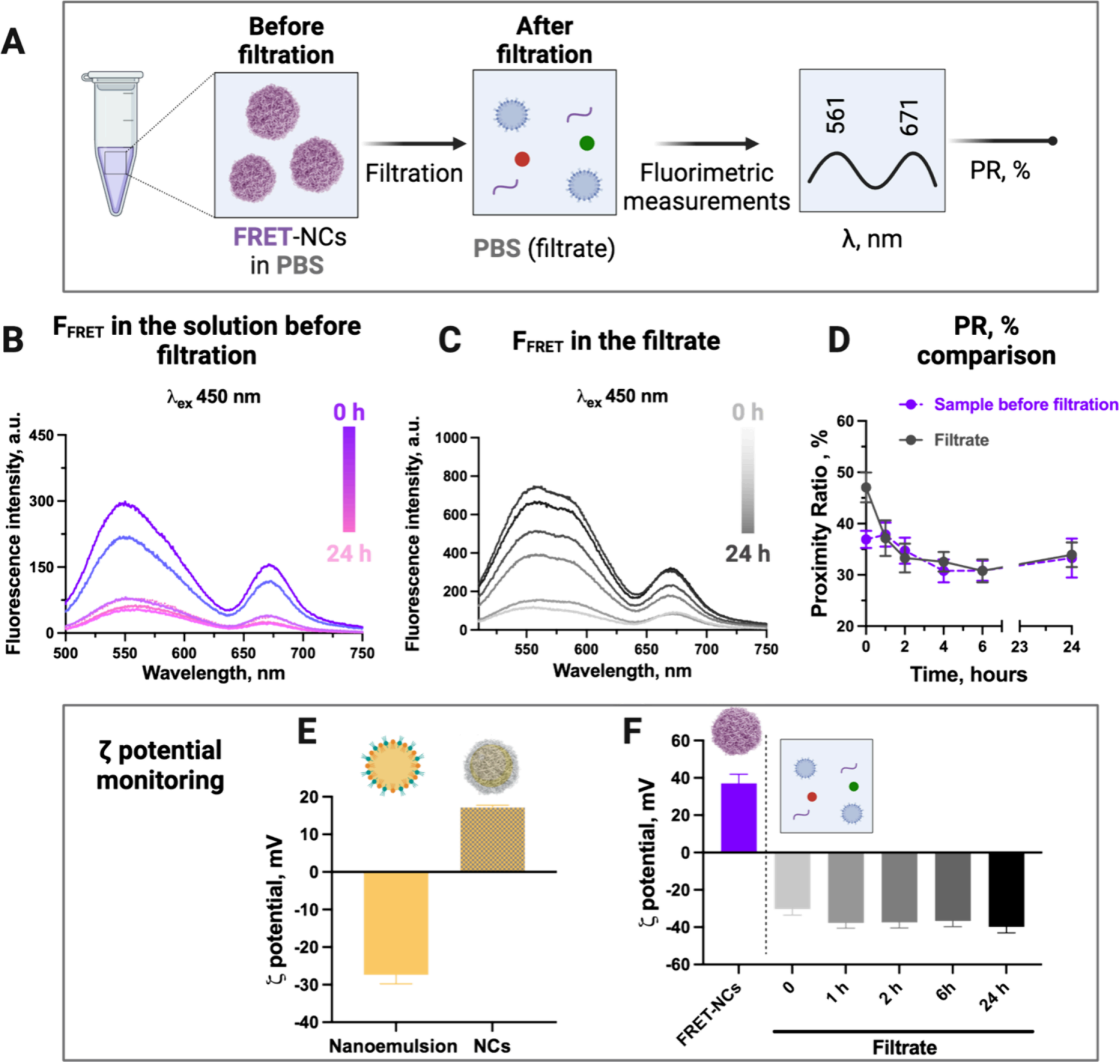
(A) Schematic representation of the release assay performed and the samples collected for analysis. (B) Fluorescent spectra of FRET-NCs in PBS during the release assay with an NC concentration at 1 mg/mL. (C) Fluorescent spectra of the filtrate were obtained by filtering the samples of the release assay. (D) Comparison between PR calculated from the spectra of the FRET-NCs in PBS and the filtered supernatant at different time points during the release assay. Comparison between the ζ potential of (E) bare NE and chitosan-coated-NCs and (F) the ζ potential of the supernatant obtained after the filtration of the FRET-NCs used for the release assay.

During the release assays described above, we also examined potential leakage from the NE through the CS shell of the NCs. To check CS fate, we monitored the fluorescent signal of sCy5@CS in both the entire release mixture (unfiltered release mixture containing PBS and sCy5@CS-DiA-NCs) and the filtrate obtained after filtering out intact NCs (Figure S13). Initially, we observed a minimal signal of sCy5@CS in the filtrate, but a significant emission peak emerged after 24 h, suggesting CS shell disruption and subsequent polymer release into the medium. This finding supports our hypothesis that the CS shell initially aids in the retention of NCs within the mucus, after which its gradual disruption could facilitate the release of the NE, allowing it to penetrate the epithelial barrier. However, since sCy5@CS-DiA-NCs did not give us information about the NE structural integrity and potential release mechanisms, we used FRET-NCs to determine if fluorophore leakage involved the intact NE or only selected components. In this case, we monitored energy transfer in both the entire release mixture (unfiltered release mixture containing PBS and FRET-NCs) and the filtrate obtained after filtering out intact NCs (Figure 4A). In the release mixture, we observed a decrease in both FRET intensity and overall fluorescence over time, likely due to a lower concentration of NCs as they adhered to the walls of the release tubes (Figure 4B). However, FRET intensity in the filtrate increased during the 24 h assay (Figure 4C), suggesting that part of the encapsulated NE may be leaking out of the CS shell and entering the release medium intact. After 24 h, the PR in the release mixture decreased from around 37 to 33%, while the filtrate decreased from 47 to 34% (Figure 4D). This decrease in PR indicates partial release of the fluorescent NE components into the medium, while the FRET signal in the filtrate points toward an intact NE.^44^ As further evidence of the presence of released NE in the filtrate, we measured the ζ potential of the filtrate, and it was consistent with those of bare NE (Figure 4E,F). Indeed, the ζ potential of all the filtrate samples collected over time was negative and in the same range of values reported for the bare NE (consisting of the addition of the organic phase to the aqueous one) we used as a control.

Overall, these studies indicated that the NE could persist intact after the loss of the CS shell. In the context of drug delivery to the brain via intranasal administration, CS contributes to mucosal adhesion by interacting with negatively charged glycoproteins in mucus, such as with mucins,^45^ whereas uncoated NE cannot exhibit this interaction.^14^ Additionally, CS enhances nanocarrier retention and permeation in the nasal mucosa compared to bare NE or free drugs.^14,46^ This property is crucial for approaches requiring prolonged release and more localized drug delivery. Furthermore, these NCs can facilitate the permeation of the NE core toward its intended target site, improving drug delivery, as demonstrated by Roger et al. for lipid NCs in the context of an intestinal epithelial barrier.^26^

### In Vitro Cellular Uptake of the NCs

Before developing a simplified in vitro barrier model, we confirmed that NCs were safe at concentrations up to 0.3 mg/mL, showing no toxicity in Calu-3 cells (Figure S14). We then tested NC internalization in Calu-3 cells using flow cytometry at 0.075, 0.15, and 0.3 mg/mL, observing that their uptake was concentration-dependent (Figure S15). Importantly, FRET was detectable by flow cytometry after internalization. Based on these results, we selected a 24 h incubation as optimal for internalization studies.

**Figure 5:**
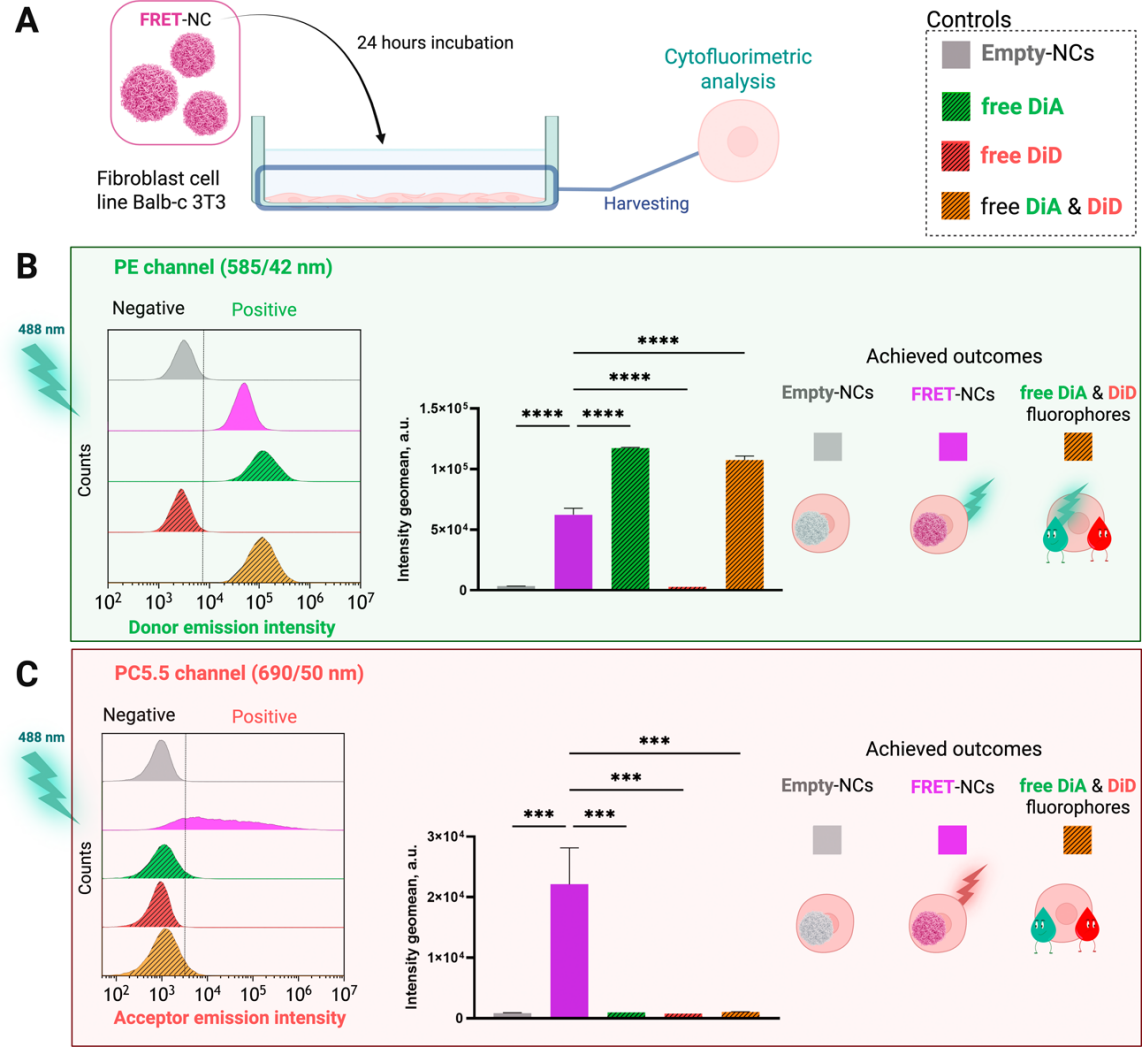
(A) Schematic representation of the internalization assay of NCs and free fluorophores in Balb-c 3T3 cells. Created with BioRender.com. Mean intracellular fluorescence intensity of Balb-c 3T3 cells incubated for 24 h with empty-NCs, FRET-NCs, free DiA, free DiD, and a mixture of free DiA and DiD was determined by flow cytometry. Cells were excited using a 488 nm laser, and the fluorescent signal was collected at the (B) PE channel (donor emission channel, 585/42 nm) and (C) PC5.5 channel (acceptor emission channel, 690/50 nm). One-way ANOVA, followed by a Dunnet multiple comparison test. Data points are statistically different with *p* ≤ 0.0003 (***) and *p* ≤ 0.0001 (****).

To ensure that fluorophores released from NCs and subsequently internalized by target cells would not colocalize in the same cellular compartment and consequently produce a false FRET signal, we evaluated the cellular uptake of free fluorophores in Balb-c 3T3 fibroblasts chosen as target cells. For this purpose, we used the same concentration of fluorophores as those encapsulated within the NCs. As shown in Figure 5, cells exposed to the mixture of the free fluorophores for 24 h did not exhibit a detectable FRET signal. This confirms that the observed FRET signal in our experiments is specifically due to intact NCs. Consequently, these results reinforce the robustness of our system for assessing NC integrity during cellular uptake and transit.

### Nanocapsule Transport across the Calu-3 Barrier Model

To evaluate the integrity and permeability of NCs across a biological barrier, we implemented a polarized cellular monolayer model based on Calu-3 cells cultured at the liquid–liquid interface (LLI). This model was characterized through TEER measurements (Figure S16), electron microscopy imaging (SEM and TEM) (Figure S17A–D), and staining techniques, all of which confirmed the formation of a robust, polarized monolayer with tight junctions and cellular differentiation (Figure S17E–G). These assessments demonstrated that the Calu-3 monolayer differentiated into mucus-producing cells and effectively mimics the in vivo epithelial barrier, providing a suitable platform for evaluating NC transport and integrity during barrier passage.

**Figure 6:**
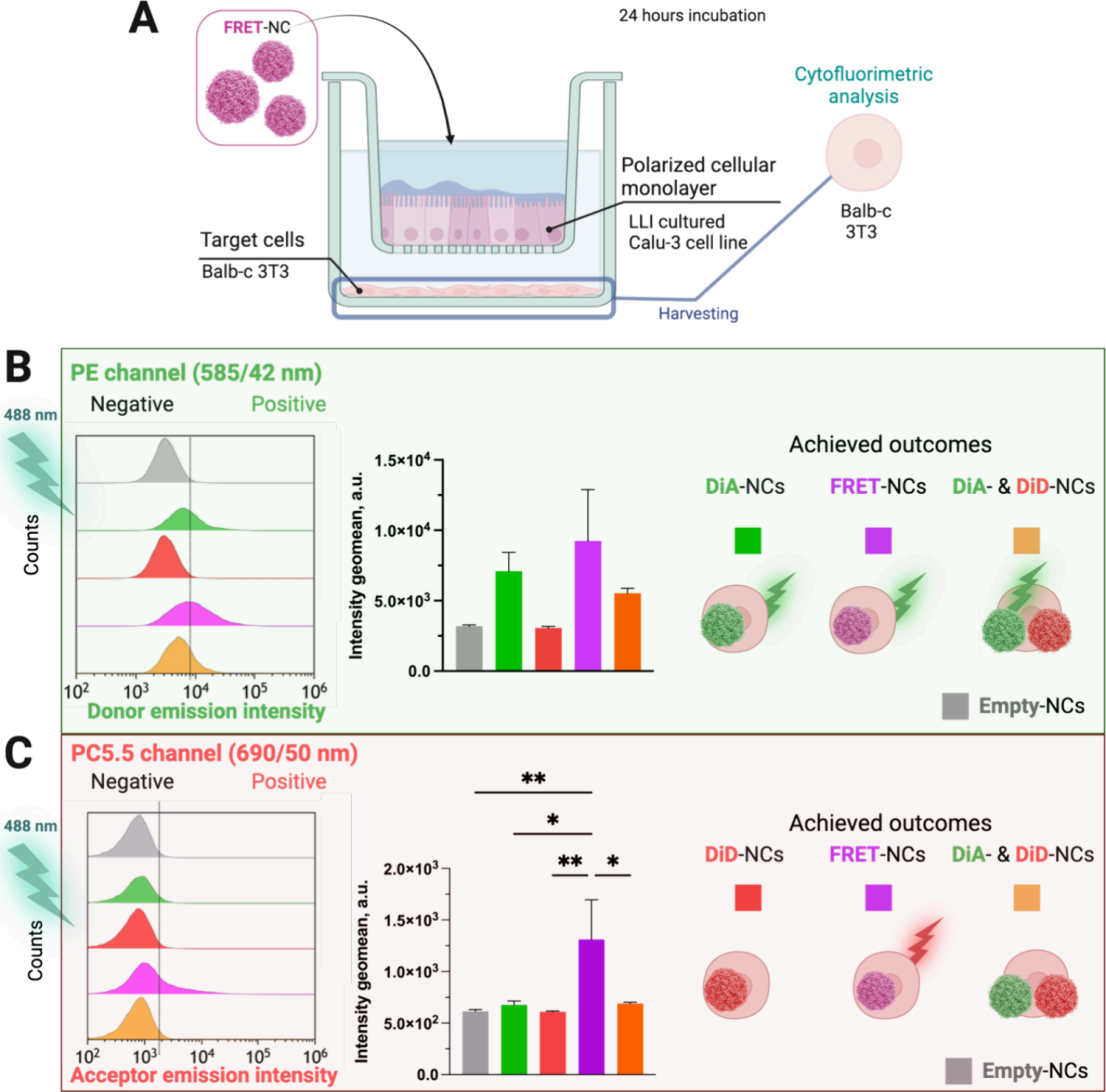
(A) Schematic representation of the Calu-3 monolayer with the Balb-c 3T3 target cells in the TW setup of the permeability assay. Created with BioRender.com. Flow cytometry histograms (left) and their corresponding geometric mean fluorescence intensity (right) of Balb-c 3T3 target cells from the basal compartment of the TW system treated with empty-NCs, DiA-NCs, DiD-NCs, FRET-NCs, and a mixture of DiA-NCs with DiD-NCs (0.3 mg/mL NC concentration, 24 h treatment) where cells were excited with a 488 nm laser, and the emission was detected (B) at the PE channel (donor emission channel, 585/42 nm), and (C) at the PC5.5 channel (acceptor emission channel, 690/50 nm). One-way ANOVA, followed by Dunnet multiple comparison test. Data points are statistically different with *p* ≤ 0.05 (*) and *p* ≤ 0.01 (**).

Calu-3 cell monolayers were cultured on TW inserts under LLI conditions and, to better resemble the in vivo situation where the cargo is required to reach the target, detector cells (fibroblasts, Balb-c 3T3 cells) were introduced in the lower compartment of the TW support (Figure 6A). 24 h after NCs were added to the apical chamber, these target cells were collected and analyzed by flow cytometry to monitor the FRET signal from the NE, the loss of which would indicate potential NE damage or disruption.

The target cells collected showed a clear FRET signal from FRET-NCs (Figures 6 and S18). The persistence of the FRET signal on the basal side after NC exposure meant the successful passage of the NE through the Calu-3 barrier model without breakdown. As we expected, in the case of target cells from the controls, which had been incubated with free fluorophores (DiA- and DiD-TPB), no FRET signal was detected (Figure S18). The absence of the FRET signal confirms that the lipophilic dyes, even if released, did not generate significant interactions or confinement capable of producing FRET in the target cells.

To further ensure that the FRET signal did not result from NE disruption and subsequent colocalization of free fluorophores within cellular compartments, besides using DiA-NCs and DiD-NCs, we also introduced a mixture of these NCs loaded with single fluorophores (DiA-NCs + DiD-NCs) (Figure 6B,C). Analysis of the target cells confirmed that only NCs with the FRET pairing produced significant FRET signals, confirming the proximity of the fluorophores within the FRET-NCs after 24 h. This result demonstrates that intact NEs successfully crossed the Calu-3 barrier, delivering their cargo to the target cells, thus underscoring their potential for transporting therapeutic payloads across cellular barriers in a stable form.

## Conclusions

In this work, we developed fluorescent NCs incorporating a FRET pair of lipophilic dyes, enabling us to monitor nanocarrier integrity through two strategies. The coencapsulation of DiA and DiD within the NCs produced a strong FRET signal, facilitating efficient NE tracking. In contrast, no FRET signal was detected when the core was loaded with DiA and the CS shell was functionalized with sCy5. This lack of signal indicates that the distance between the fluorophores exceeded 10 nm, providing valuable insights into the structural arrangement of the NCs. To further investigate their behavior, we conducted release assays of the FRET-NCs obtained by fluorophore coencapsulation, highlighting the potential of the NE to diffuse through the CS shell. This diffusion capability could enhance epithelium penetration following mucosal adhesion provided by CS, allowing for effective drug delivery in brain-targeted therapies. Our study utilized a simplified respiratory model with Calu-3 cells to replicate nasal mucosal behavior, where the FRET signal served as a reliable marker for monitoring NE integrity during permeability experiments. Remarkably, target cells beneath the epithelial monolayer that were treated with the FRET-NCs exhibited a FRET signal, confirming that the NE can traverse the epithelium while remaining intact. Given their straightforward and flexible preparation, these NCs hold significant promise for applications in nasal brain delivery, including bioimaging, therapeutic, and theragnostic strategies.

## Supporting Information Available

General procedures; establishment and characterization of a simplified in vitro nasal mucosal barrier model using Calu-3 cells; simplified in vitro nasal mucosal barrier model with Calu-3; morphological, histological, and histochemical structure study of the Calu-3 model barrier; cell junction protein immunolabeling; supplementary figures and tables; CS functionalization with sCy5-NHS ester; counterion substitution of DiA and DiD fluorophores; determination of encapsulation efficiency and fluorophore loading; release studies; permeability/transcellular transport studies through the Calu-3 model barrier; preparation of the fluorophore-loaded NCs; excitation and emission spectra of the fluorophores; encapsulation efficiency and fluorophore loading; zeta potential, size, and morphology of the NCs; emission spectra of sCy5@NCs and sCy5@CS-DiA-NCs; FRET signal evaluation by fluorimetry and flow cytometry; NCs stability and NE diffusion through the CS shell in release assays; in vitro cytotoxicity of the NCs; in vitro cellular uptake of the NCs; establishment and characterization of a simplified in vitro nasal mucosal barrier model using Calu-3 cells; nanocapsule transport across the Calu-3 barrier model; and references (PDF)

## Abbreviations

CS: chitosan
DiA: 4-Di-16-ASP (4-(4-(dihexadecylamino)styryl)-*N*-methylpyridinium
DiD: (1,1′-dioctadecyl-3,3,3′,3′-tetramethylindodicarbocyanine
FRET: Förster resonance energy transfer
NC: nanocapsule
NE: nanoemulsion
PC: perchlorate
sCy5: sulfo-Cyanine 5
TPB: tetraphenylborate
TW: Transwell

## References

[ref1] Sarvaiya J.; Agrawal Y. K. Chitosan as a Suitable Nanocarrier Material for Anti-Alzheimer Drug Delivery. Int. J. Biol. Macromol. 2015, 72, 454–465. 10.1016/j.ijbiomac.2014.08.052. 25199867

[ref2] Kozlovskaya L.; Abou-Kaoud M.; Stepensky D. Quantitative Analysis of Drug Delivery to the Brain via Nasal Route. J. Controlled Release 2014, 189, 133–140. 10.1016/j.jconrel.2014.06.053. 24997277

[ref3] Agrawal M.; Saraf S.; Saraf S.; Antimisiaris S. G.; Chougule M. B.; Shoyele S. A.; Alexander A. Nose-to-Brain Drug Delivery: An Update on Clinical Challenges and Progress towards Approval of Anti-Alzheimer Drugs. J. Controlled Release 2018, 281, 139–177. 10.1016/j.jconrel.2018.05.011. 29772289

[ref4] Silva S.; Bicker J.; Falcão A.; Fortuna A. Air-Liquid Interface (ALI) Impact on Different Respiratory Cell Cultures. Eur. J. Pharm. Biopharm. 2023, 184, 62–82. 10.1016/j.ejpb.2023.01.013. 36696943

[ref5] Li J.; Cai C.; Li J.; Li J.; Li J.; Sun T.; Wang L.; Wu H.; Yu G. Chitosan-Based Nanomaterials for Drug Delivery. Molecules 2018, 23 (10), 2661 10.3390/molecules23102661. 30332830 PMC6222903

[ref6] Wang Q. Z.; Chen X. G.; Liu N.; Wang S. X.; Liu C. S.; Meng X. H.; Liu C. G. Protonation Constants of Chitosan with Different Molecular Weight and Degree of Deacetylation. Carbohydr. Polym. 2006, 65 (2), 194–201. 10.1016/j.carbpol.2006.01.001.

[ref7] Illum L.; Watts P.; Fisher A. N.; Hinchcliffe M.; Norbury H.; Jabbal-Gill I.; Nankervis R.; Davis S. S. Intranasal Delivery of Morphine. J. Pharmacol. Exp. Ther. 2002, 301 (1), 391–400. 10.1124/jpet.301.1.391. 11907197

[ref8] Gulati N.; Nagaich U.; Saraf S. A. Intranasal Delivery of Chitosan Nanoparticles for Migraine Therapy. Sci. Pharm. 2013, 81 (3), 843–854. 10.3797/scipharm.1208-18. 24106677 PMC3791944

[ref9] Ahmad E.; Feng Y.; Qi J.; Fan W.; Ma Y.; He H.; Xia F.; Dong X.; Zhao W.; Lu Y.; Wu W. Evidence of Nose-to-Brain Delivery of Nanoemulsions: Cargoes but Not Vehicles. Nanoscale 2017, 9 (3), 1174–1183. 10.1039/C6NR07581A. 28009915

[ref10] Aranaz I.; Alcántara A. R.; Civera M. C.; Arias C.; Elorza B.; Caballero A. H.; Acosta N. Chitosan: An Overview of Its Properties and Applications. Polymers 2021, 13 (19), 3256 10.3390/polym13193256. 34641071 PMC8512059

[ref11] Rodrigues S.; Dionísio M.; López C. R.; Grenha A. Biocompatibility of Chitosan Carriers with Application in Drug Delivery. J. Funct. Biomater. 2012, 3 (3), 615–641. 10.3390/jfb3030615. 24955636 PMC4030999

[ref12] Garg U.; Chauhan S.; Nagaich U.; Jain N. Current Advances in Chitosan Nanoparticles Based Drug Delivery and Targeting. Adv. Pharm. Bull. 2019, 9 (2), 195–204. 10.15171/apb.2019.023. 31380245 PMC6664124

[ref13] Erdő F.; Bors L. A.; Farkas D.; Bajza Á.; Gizurarson S. Evaluation of Intranasal Delivery Route of Drug Administration for Brain Targeting. Brain Res. Bull. 2018, 143, 155–170. 10.1016/j.brainresbull.2018.10.009. 30449731

[ref14] Duarte J. L.; Di Filippo L. D.; Azevedo Vilella K. J.; Paes Dutra J. A.; Ribeiro D. M.; Freitas da Silva M.; Ivo de Medeiros A.; Chorilli M. Chitosan-Coated Nanoemulsion for Intranasal Administration Increases Temozolomide Mucosal Permeation, Cellular Uptake, and In Vitro Cytotoxicity in Glioblastoma Multiforme Cells. J. Drug Delivery Sci. Technol. 2024, 102, 106390 10.1016/j.jddst.2024.106390.

[ref15] Casettari L.; Illum L. Chitosan in Nasal Delivery Systems for Therapeutic Drugs. J. Controlled Release 2014, 190, 189–200. 10.1016/j.jconrel.2014.05.003. 24818769

[ref16] Wibel R.; Braun D. E.; Hämmerle L.; Jörgensen A. M.; Knoll P.; Salvenmoser W.; Steinbring C.; Bernkop-Schnürch A. In Vitro Investigation of Thiolated Chitosan Derivatives as Mucoadhesive Coating Materials for Solid Lipid Nanoparticles. Biomacromolecules 2021, 22 (9), 3980–3991. 10.1021/acs.biomac.1c00776. 34459197 PMC8441978

[ref17] Azmi N. A. N.; Elgharbawy A. A. M.; Motlagh S. R.; Samsudin N.; Salleh H. M. Nanoemulsions: Factory for Food, Pharmaceutical and Cosmetics. Processes 2019, 7 (9), 617 10.3390/pr7090617.

[ref18] Solans C.; Izquierdo P.; Nolla J.; Azemar N.; Garcia-Celma M. J. Nano-Emulsions. Curr. Opin. Colloid Interface Sci. 2005, 10 (3–4), 102–110. 10.1016/j.cocis.2005.06.004.

[ref19] Sheth T.; Seshadri S.; Prileszky T.; Helgeson M. E. Multiple Nanoemulsions. Nat. Rev. Mater. 2020, 5 (3), 214–228. 10.1038/s41578-019-0161-9.

[ref20] De Matteis L.; Alleva M.; Serrano-Sevilla I.; García-Embid S.; Stepien G.; Moros M.; De La Fuente J. M. Controlling Properties and Cytotoxicity of Chitosan Nanocapsules by Chemical Grafting. Mar. Drugs 2016, 14 (10), 175 10.3390/md14100175. 27706041 PMC5082323

[ref21] De Matteis L.; Jary D.; Lucía A.; García-Embid S.; Serrano-Sevilla I.; Pérez D.; Ainsa J. A.; Navarro F. P.; de la Fuente J. M. New Active Formulations against M. Tuberculosis: Bedaquiline Encapsulation in Lipid Nanoparticles and Chitosan Nanocapsules. Chem. Eng. J. 2018, 340, 181–191. 10.1016/j.cej.2017.12.110.

[ref22] Coya J. M.; De Matteis L.; Giraud-Gatineau A.; Biton A.; Serrano-Sevilla I.; Danckaert A.; Dillies M. A.; Gicquel B.; De La Fuente J. M.; Tailleux L. Tri-Mannose Grafting of Chitosan Nanocarriers Remodels the Macrophage Response to Bacterial Infection. J. Nanobiotechnology 2019, 17 (1), 15 10.1186/s12951-018-0439-x. 30683129 PMC6346558

[ref23] Casadomé-Perales Á.; De Matteis L.; Alleva M.; Infantes-Rodríguez C.; Palomares-Pérez I.; Saito T.; Saido T. C.; Esteban J. A.; Nebreda A. R.; De La Fuente J. M.; Dotti C. G. Inhibition of P38 MAPK in the Brain through Nasal Administration of P38 Inhibitor Loaded in Chitosan Nanocapsules. Nanomedicine 2019, 14 (18), 2409–2422. 10.2217/nnm-2018-0496. 31456488

[ref24] Forte E.; Fiorenza D.; Torino E.; Di Polidoro A. C.; Cavaliere C.; Netti P. A.; Salvatore M.; Aiello M. Radiolabeled PET/MRI Nanoparticles for Tumor Imaging. J. Clin. Med. 2020, 9 (1), 89 10.3390/jcm9010089. PMC7019574 31905769

[ref25] Bastiat G.; Pritz C. O.; Roider C.; Fouchet F.; Lignières E.; Jesacher A.; Glueckert R.; Ritsch-Marte M.; Schrott-Fischer A.; Saulnier P.; Benoit J. P. A New Tool to Ensure the Fluorescent Dye Labeling Stability of Nanocarriers: A Real Challenge for Fluorescence Imaging. J. Controlled Release 2013, 170 (3), 334–342. 10.1016/j.jconrel.2013.06.014. 23792117

[ref26] Roger E.; Gimel J. C.; Bensley C.; Klymchenko A. S.; Benoit J. P. Lipid Nanocapsules Maintain Full Integrity after Crossing a Human Intestinal Epithelium Model. J. Controlled Release 2017, 253, 11–18. 10.1016/j.jconrel.2017.03.005. 28274740

[ref27] Chen T.; He B.; Tao J.; He Y.; Deng H.; Wang X.; Zheng Y. Application of Förster Resonance Energy Transfer (FRET) Technique to Elucidate Intracellular and In Vivo Biofate of Nanomedicines. Adv. Drug Delivery Rev. 2019, 143, 177–205. 10.1016/j.addr.2019.04.009. 31201837

[ref28] Shrestha D.; Jenei A.; Nagy P.; Vereb G.; Szöllősi J. Understanding FRET as a Research Tool for Cellular Studies. Int. J. Mol. Sci. 2015, 16 (4), 6718–6756. 10.3390/ijms16046718. 25815593 PMC4424985

[ref29] Arai Y.; Nagai T. Extensive Use of FRET in Biological Imaging. J. Electron Microsc. (Tokyo). 2013, 62 (4), 419–428. 10.1093/jmicro/dft037. 23797967

[ref30] Valdez S.; Robertson M.; Qiang Z. Fluorescence Resonance Energy Transfer Measurements in Polymer Science: A Review. Macromol. Rapid Commun. 2022, 43 (24), 2200421 10.1002/marc.202200421. 35689335

[ref31] Kaeokhamloed N.; Legeay S.; Roger E. FRET as the Tool for in Vivo Nanomedicine Tracking. J. Controlled Release 2022, 349, 156–173. 10.1016/j.jconrel.2022.06.048. 35779657

[ref32] Kilin V. N.; Anton H.; Anton N.; Steed E.; Vermot J.; Vandamme T. F.; Mely Y.; Klymchenko A. S. Counterion-Enhanced Cyanine Dye Loading into Lipid Nano-Droplets for Single-Particle Tracking in Zebrafish. Biomaterials 2014, 35 (18), 4950–4957. 10.1016/j.biomaterials.2014.02.053. 24661553

[ref33] Klymchenko A. S.; Liu F.; Collot M.; Anton N. Dye-Loaded Nanoemulsions: Biomimetic Fluorescent Nanocarriers for Bioimaging and Nanomedicine. Adv. Healthc. Mater. 2021, 10 (1), 2001289 10.1002/adhm.202001289. 33052037

[ref34] Sabourian P.; Yazdani G.; Ashraf S. S.; Frounchi M.; Mashayekhan S.; Kiani S.; Kakkar A. Effect of Physico-Chemical Properties of Nanoparticles on Their Intracellular Uptake. Int. J. Mol. Sci. 2020, 21 (21), 8019 10.3390/ijms21218019. 33126533 PMC7662525

[ref35] Bannunah A. M.; Vllasaliu D.; Lord J.; Stolnik S. Mechanisms of Nanoparticle Internalization and Transport across an Intestinal Epithelial Cell Model: Effect of Size and Surface Charge. Mol. Pharmaceutics 2014, 11 (12), 4363–4373. 10.1021/mp500439c. 25327847

[ref36] Bej R.; Haag R. Mucus-Inspired Dynamic Hydrogels: Synthesis and Future Perspectives. J. Am. Chem. Soc. 2022, 144 (44), 20137–20152. 10.1021/jacs.1c13547. 36074739 PMC9650700

[ref37] Alghareeb S.; Asare-Addo K.; Conway B. R.; Adebisi A. O. PLGA Nanoparticles for Nasal Drug Delivery. J. Drug Delivery Sci. Technol. 2024, 95, 105564 10.1016/j.jddst.2024.105564.

[ref38] Nojoki F.; Ebrahimi-Hosseinzadeh B.; Hatamian-Zarmi A.; Khodagholi F.; Khezri K. Design and Development of Chitosan-Insulin-Transfersomes (Transfersulin) as Effective Intranasal Nanovesicles for the Treatment of Alzheimer’s Disease: In Vitro, in Vivo, and Ex Vivo Evaluations. Biomed. Pharmacother. 2022, 153, 113450 10.1016/j.biopha.2022.113450. 36076565

[ref39] Charron D. M.; Zheng G. Nanomedicine Development Guided by FRET Imaging. Nano Today 2018, 18, 124–136. 10.1016/j.nantod.2017.12.006.

[ref40] Qu S.; Liu C.; Liu Q.; Wu W.; Du B.; Wang J. Solvent Effect on FRET Spectroscopic Ruler. J. Chem. Phys. 2018, 148 (12), 123331 10.1063/1.5004205. 29604875

[ref41] Maag P. H.; Feist F.; Frisch H.; Roesky P. W.; Barner-Kowollik C. Förster Resonance Energy Transfer within Single Chain Nanoparticles. Chem. Sci. 2024, 15 (14), 5218–5224. 10.1039/D3SC06651G. 38577362 PMC10988607

[ref42] Picola I. P. D.; Busson K. A. N.; Casé A. H.; Nasário F. D.; Tiera V. A. d. O.; Taboga S. R.; Neto J. R.; Tiera M. J. Effect of Ionic Strength Solution on the Stability of Chitosan-DNA Nanoparticles. J. Exp. Nanosci. 2013, 8 (5), 703–716. 10.1080/17458080.2011.602120.

[ref43] Pederzoli F.; Tosi G.; Vandelli M. A.; Belletti D.; Forni F.; Ruozi B. Protein Corona and Nanoparticles: How Can We Investigate On? *Wiley Interdiscip*. Rev. Nanomedicine Nanobiotechnology 2017, 9 (6), e1467 10.1002/wnan.1467. 28296346

[ref44] Swider E.; Maharjan S.; Houkes K.; Van Riessen N. K.; Figdor C.; Srinivas M.; Tagit O. Förster Resonance Energy Transfer-Based Stability Assessment of PLGA Nanoparticles in Vitro and in Vivo. ACS Appl. Bio Mater. 2019, 2 (3), 1131–1140. 10.1021/acsabm.8b00754. PMC6428147 30906926

[ref45] Groo A. C.; Lagarce F. Mucus Models to Evaluate Nanomedicines for Diffusion. Drug Discovery Today 2014, 19 (8), 1097–1108. 10.1016/j.drudis.2014.01.011. 24491319

[ref46] Dong W.; Ye J.; Zhou J.; Wang W.; Wang H.; Zheng X.; Yang Y.; Xia X.; Liu Y. Comparative Study of Mucoadhesive and Mucus-Penetrative Nanoparticles Based on Phospholipid Complex to Overcome the Mucus Barrier for Inhaled Delivery of Baicalein. Acta Pharm. Sin. B 2020, 10 (8), 1576–1585. 10.1016/j.apsb.2019.10.002. 32963951 PMC7488487

