# Supplementary material for: Förster Resonance Energy Transfer (FRET) Demonstrates In Vitro Chitosan-Coated Nanocapsules Suitability for Intranasal Brain Delivery
Source: ACS Appl Mater Interfaces. 2025 Apr 28;17(18):26348–60. doi: 10.1021/acsami.5c01920 (PMC12067367; doi:10.1021/acsami.5c01920)
Supplement: Supplementary file 1 — am5c01920_si_001.pdf [file am5c01920_si_001.pdf]

Supporting Information

Förster Resonance Energy Transfer (FRET) demonstrates *in vitro* chitosan-coated nanocapsules suitability for intranasal brain delivery

Maria Alleva [a], Zsuzsa Baranyai [a,b], Natalia Esteban-Pérez [a,b], Pablo Martínez-Vicente [a,c], Rafael Martín-Rapún [a,b], María Moros [a,b], Jesús Martínez de la Fuente [a,b]**

[a] Instituto de Nanociencia y Materiales de Aragón (INMA), CSIC-Universidad de Zaragoza, Zaragoza 50009, Spain

[b] Centro de Investigación Biomédica en Red de Bioingeniería, Biomateriales y Nanomedicina (CIBER-BBN), Madrid 28029, Spain

[c] Departamento de Bioquímica y Biología Molecular y Celular, Facultad de Ciencias de la Salud y el Deporte, Universidad de Zaragoza, Huesca 22002, Spain

* Email: zsuzsa.baranyai@csic.es

* Email: j.m.fuente@csic.es

Table of Contents

General procedures 3

Establishment and characterization of a simplified *in vitro* nasal mucosal barrier model using Calu-3 cells 5

Simplified *in vitro* nasal mucosal barrier model with Calu-3 5

Morphological, histological and histochemical structure study of the Calu-3 model barrier ... 5

Cell junction protein immunolabeling 6

Supplementary figures and tables 7

CS functionalization with sCy5-NHS ester 7

Counterion substitution of DiA and DiD fluorophores 8

Encapsulation efficiency and fluorophore loading 9

Release studies 9

Permeability/transcellular transport studies through Calu-3 model barrier 10

Preparation of the fluorophore-loaded NCs 11

Excitation and emission spectra of the fluorophores 12

Encapsulation efficiency and fluorophore loading 13

Zeta potential, size, and morphology of the NCs 13

Emission spectra of sCy5@CS-NCs and sCy5@CS-DiA-NCs 15

FRET signal evaluation by fluorimetry and flow cytometry 16

NCs stability and NE diffusion through the CS shell in release assays 17

In vitro cytotoxicity of the NCs 18

In vitro cellular uptake of the NCs 19

Establishment and characterization of a simplified *in vitro* nasal mucosal barrier model using Calu-3 cells 20

NC transport across the Calu-3 barrier model 23

References 24

General procedures

Characterization of sCy5@chitosans by IR, NMR and UV-VIS

FTIR-ATR spectroscopy of sCy5@chitosans was performed on a Jasco FT-IR 4100 (Jasco Inc., Easton, MD, US) spectrometer recorded in the 400−4000 cm^{-1} wavenumber range, at a resolution of 4 cm^{-1}. Solid samples were measured without further preparation. The spectra were represented with GraphPad Prism version 8.0 (GraphPad Software Inc., Boston, MA, USA), characteristic bands are reported in cm^{-1} (Figure S5B).

sCy5@chitosans were dissolved in D_2O with 1% TFA (v/v). ^{1}H-NMR spectra were recorded at room temperature using a Bruker Avance 400 MHz NMR spectrometer (Bruker Corporation, Billerica, MA, US). Chemical shifts are given in ppm relative to the peak of 3-(trimethylsilyl)propionic-2,2,3,3-d4 acid sodium salt, which was used as internal reference. Spectra were processed with TopSpin 4.2.0 (Bruker Corporation) (Figure S5C).

SEM of nanocapsules

For scanning electron microscopy (SEM) sample observation was carried out using an F-50 Inspect (FEI Company, Thermo Fisher Scientific) at 10 kV (Figure S8A, B). The sample was deposited on a clean glass coverslip (10 mm in diameter) and left to dry for 5 min, then an equal volume of fixing solution (4% PFA in phosphate buffer) was added and left for 30 min. After this, the sample was washed three times with distilled water for 1 min each, then post-fixed with 2% OsO_4 (4% in water) for 1 h at room temperature and washed three times with water for 1 min each. This was followed by several dehydration cycles of 10 min each in ethanol with increasing concentrations (30-50-70-100%). The sample was then dried using hexamethyldisilazane (HMDS, 98%): EtOH 100% (50:50, v/v) for 10 min. Finally, 100% HMDS was added and left for 30 min. The coverslip was then air-dried in a fume hood. The coverslip was mounted on an aluminum pin with carbon tape and coated with 14 nm of Pd.

In vitro cytotoxicity of the nanocapsules

Calu-3 cells were harvested when 80-100% confluence was reached using trypsin-EDTA, collected, and centrifuged at 1200 rpm for 6 min. The cell pellet was resuspended in cMEM medium, the cells were counted with a haemocytometer and plated on 96-well plates (TPP, Techno Plastic Products AG, Trasadingen, Switzerland) 2 days before the experiment. Cells were seeded in the plates at the concentration of 40 000 cells/100 µL/well in cMEM medium and incubated for 48 h (37 °C, 5% CO_2). After incubation, cells were washed twice with PBS and treated with the nanocapsules in incomplete MEM in 200 µL final volume. A stock solution of nanocapsules of a concentration of 10

mg/mL in water was used for preparing the serial dilution of treating concentrations (0.04-0.6 mg/mL) in MEM medium. Control cells were treated with medium only or 6% (v/v) water-containing medium (equivalent water content of the highest concentrations of nanocapsule treating solution). After 24 h of incubation, cell viability was determined with MTT assay. Cells were washed twice with PBS then 100 μL 0.5 mg/mL MTT ((4,5-dimethylthiazol-2-yl)-2,5-diphenyltetrazolium bromide, Thermo Fisher Scientific) solution was added to each well and during 2 h of incubation (37 °C, 5% CO_2), purple formazan crystals were formed by mitochondrial dehydrogenase enzyme present in the living cells. After incubation, cells were centrifuged for 25 min at 2500 rpm and the supernatant was removed. The obtained formazan crystals were dissolved in DMSO, and the optical density (OD) of the samples was determined at 570 and 630 nm using a BioTek Synergy H1 microplate reader. The OD_{630} values were subtracted from the OD_{570} values. The percentage of cell viability was calculated using the following equation: viability (%) = 100 × ($OD_{treated}$/$OD_{control}$), where $OD_{treated}$ and $OD_{control}$ correspond to the optical densities of treated and control cells, respectively. In each case, two independent experiments were carried out with four parallel measurements. Results were presented using GraphPad Prism (version 8.0) software (Figure S14).

Cellular uptake studies by flow cytometry

Calu-3 cells were plated on 24-well plates (TPP) 2 days before the experiment at the concentration of 100 000 cells/500 μL/well in cMEM medium and incubated for 48 h (37 °C, 5% CO_2). After incubation, cells were washed twice with DPBS and treated with the nanocapsules in 500 μL final volume with treating concentrations of 0.075, 0.15, 0.3 mg/mL. Control cells were treated with medium only. After 3 h of incubation cells were washed twice with DPBS, the supernatant was removed, and 100 μL of trypsin-EDTA was added to the cells. After 15 min of incubation at 37 °C, trypsin was inactivated by 800 μL complete medium. Then cells were transferred from the plate to tubes, centrifuged (1500 rpm, 5 min), and the supernatant was removed. After this procedure, cells were resuspended in 150 μL of 10% FBS containing PBS, and their intracellular fluorescence intensity was measured on a CytoFLEX flow cytometer using a 488 nm laser, PE channel for the detection of DiA fluorescence (emission at 585/42 nm) and PC5.5 channel the detection of DiD and sCy5 fluorescence (emission at 690/50 nm). Data was analyzed with CytExpert 2.4 software and Kaluza 2.1 software. Live cells were first gated on forward scatter area (FSC-A) vs side scatter-area (SSC-A), followed by side scatter-height (SSC-H) vs SSC-A to gate on single cells, before designating fluorescence negative and positive population gates using a histogram (Figure S4). All experiments were performed in triplicates. Data was presented using GraphPad Prism (version 8.0) software.

Establishment and characterization of a simplified *in vitro* nasal mucosal barrier model using Calu-3 cells

Simplified *in vitro* nasal mucosal barrier model with Calu-3

Cells were seeded onto membrane filters (Transwell® inserts, polyethylene terephthalate (PET), 3.0 μm pore size, 1.12 cm^2 growth area) in Transwell® cell culture chambers (Product Number 3462, Corning Costar, Cambridge, MA, USA) at a density of 400 000 cell/500 μL/insert (equal to 357 000 cells/cm^2). Every two days for 18 days, the culture medium from the apical and basal chambers was removed, cells were washed twice with 1 mL PBS, both chambers were filled with 1 mL PBS, and transepithelial electrical resistance (TEER) was measured to monitor monolayer formation on the inserts using a Millicell®-ER-2 system (Millipore Corporation, Billerica, MA). Then, PBS was replaced with fresh cMEM (0.5 mL per insert (apical part) and 1 mL per well (basal part)) for further culturing. After 10-12 days in culture, cell monolayers with TEER values over 500 $\Omega \cdot cm^2$ were used for experiments (Figure S16).

Morphological, histological and histochemical structure study of the Calu-3 model barrier

The morphological characterization of Calu-3 cells was performed through the examination of the cellular layer with SEM and TEM (Figure S17A-D). For SEM analysis, Calu-3 cells were seeded and cultured on Transwell® inserts as described above. Samples were washed with PBS twice, then 250 μL 4% glutaraldehyde in 0.2 M sodium cacodylate buffer (pH 7.2, adjusted with HCl) was added, and samples were incubated for 2 h at 4 °C. After removing the supernatant, samples were washed three times with 250 μL 2% glutaraldehyde in 0.1 M sodium cacodylate buffer, pH 7.2. Then, dehydration was carried out at room temperature in a graded series of MeOH: 30 % (in water), 50%, 70%, 100%, and anhydrous MeOH (2 times, for 5 min each step). The samples were then dried at room temperature using a graded series of HMDS/absolute EtOH, v/v: 1%, 10%, 25%, 50%, and 100% for 10 min for each step. The resulting samples were placed over a glass slide on conductive carbon tape and left for the HMDS to evaporate completely. Finally, samples were sputter-coated with 14 nm of palladium and viewed under an F-50 Inspect SEM instrument using 10 kV of accelerating voltage.

For TEM analysis cells were seeded and cultured on Transwell® inserts as described above. Samples were fixed with 1 mL 2.5% glutaraldehyde/2% PFA in 0.1 M phosphate buffer, pH 7.4, at room temperature for 15 min, then at 4 °C for 1 h. The inserts were postfixed in 2% OsO_4 for 1 h at room temperature and stained in 2% uranyl acetate in the dark for 2 h at 4 °C. Then, samples were rinsed in distilled water, dehydrated in EtOH, and infiltrated overnight in Durcupan resin (Fluka, Sigma-Aldrich). Following polymerization, ultra-thin sections (0.08 μm) were cut with an Ultracut

UC-6 (Leica microsystems, Wetzlar, Germany), stained with lead citrate (Reynolds solution), and examined with a Tecnai T20 instrument.

Alcian blue (AB) staining was used to verify mucus production (Figure S17E). Calu-3 cells were seeded and cultured on Transwell® inserts as described above, washed twice with PBS, then samples were fixed in 4% PFA. The sample processing was carried out using a Tissue-Tek Xpress x50 tissue processor (Sakura Finetek USA Inc., Torrance, CA, USA). Samples were embedded in paraffin, the block preparation was performed with a Leica EG1150 embedding station (Leica Biosystems, Wetzlar, Germany). Once the blocks were cooled, they were cut into 3 µm thick sections using a Leica RM2255 (Leica Biosystems) rotary microtome and left to dry overnight at 37 °C. For the AB staining the entire process was conducted on the Leica ST5020 staining station (Leica Biosystems). The samples were deparaffinized in xylene for 10 min and rehydrated in a series of EtOH (100%, 100%, 96%, 70%, 5 min each) and distilled water (1 min). Subsequently, they were immersed in a solution of AB at pH 2.5 for 30 min, washed with water for 5 min, and stained with Nuclear Fast Red (BioOptica Milano S.p.A, Milano, Italy) for 2 min. After washing with water for 2 min, the samples were dehydrated by immersion in ascending EtOH solutions (70%, 96%, 100%, 100%, 15 s each) and cleared with 2 immersions of 15 s each in xylene. Finally, they were mounted with Leica CV Mounting Medium (Leica Biosystems) on glass slides. Samples were visualized using an inverted fluorescence microscope (Nikon Eclipse Ti-E, Nikon Instruments Inc., Tokyo, Japan) in bright field mode.

Cell junction protein immunolabeling

To confirm the formation of a tight cell monolayer, Calu-3 cells were seeded at a density of 300 000 cells/500 µL/well and cultured on glass coverslips in a 24-well plate (TPP) for 5 days. After washing with PBS, cells were fixed using 4% PFA for 20 minutes at 4 °C and then washed with PBS twice. Cell membranes were permeabilized with 0.1 % Triton X-100 in PBS for 10 min, then blocked with 1% BSA in PBS during 1 h at room temperature. Lastly, cells were incubated for 2 h at room temperature with the mouse ZO-1 monoclonal antibody conjugated with Alexa Fluor 488, diluted 1:100 in a solution of 0.1% BSA in PBS. Two rinses with 0.1% Tween® 20 in PBS were then performed, and nuclei were stained with a solution of DAPI (3 µM in PBS, 200 µL/well). The coverslips were mounted with a drop of ProLong™ Diamond, then sealed with nail polish to prevent drying, and samples were stored in the dark at 4 °C. Images were obtained using an inverted fluorescence microscope (Nikon Eclipse Ti-E) using the following settings: for DAPI: excitation: 387/11 nm, emission 447/60 nm; for Alexa Fluor 488 excitation: 472/30 nm, emission: 530/35 nm (Figure S17F,G).

Supplementary figures and tables

CS functionalization with sCy5-NHS ester

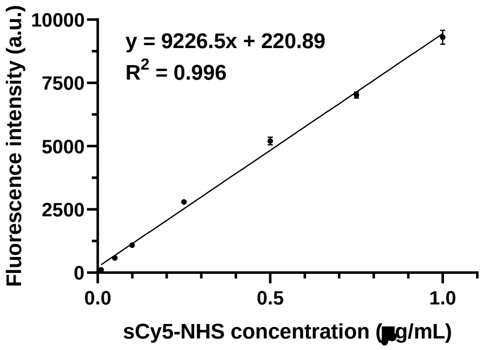

Figure S1. Calibration curve of sCy5-NHS ester for the characterization of sCy5@CSs

Table S1. Theoretical and measured substitution degree of sCy5@CSs

sCy5@CS code	Theoretical substitution degree (%)	Measured substitution degree (%)
sCy5@CS-5	5	4.41 ± 0.03
sCy5@CS-1	1	1.32 ± 0.00
sCy5@CS-0.2	0.2	0.19 ± 0.02

Counterion substitution of DiA and DiD fluorophores

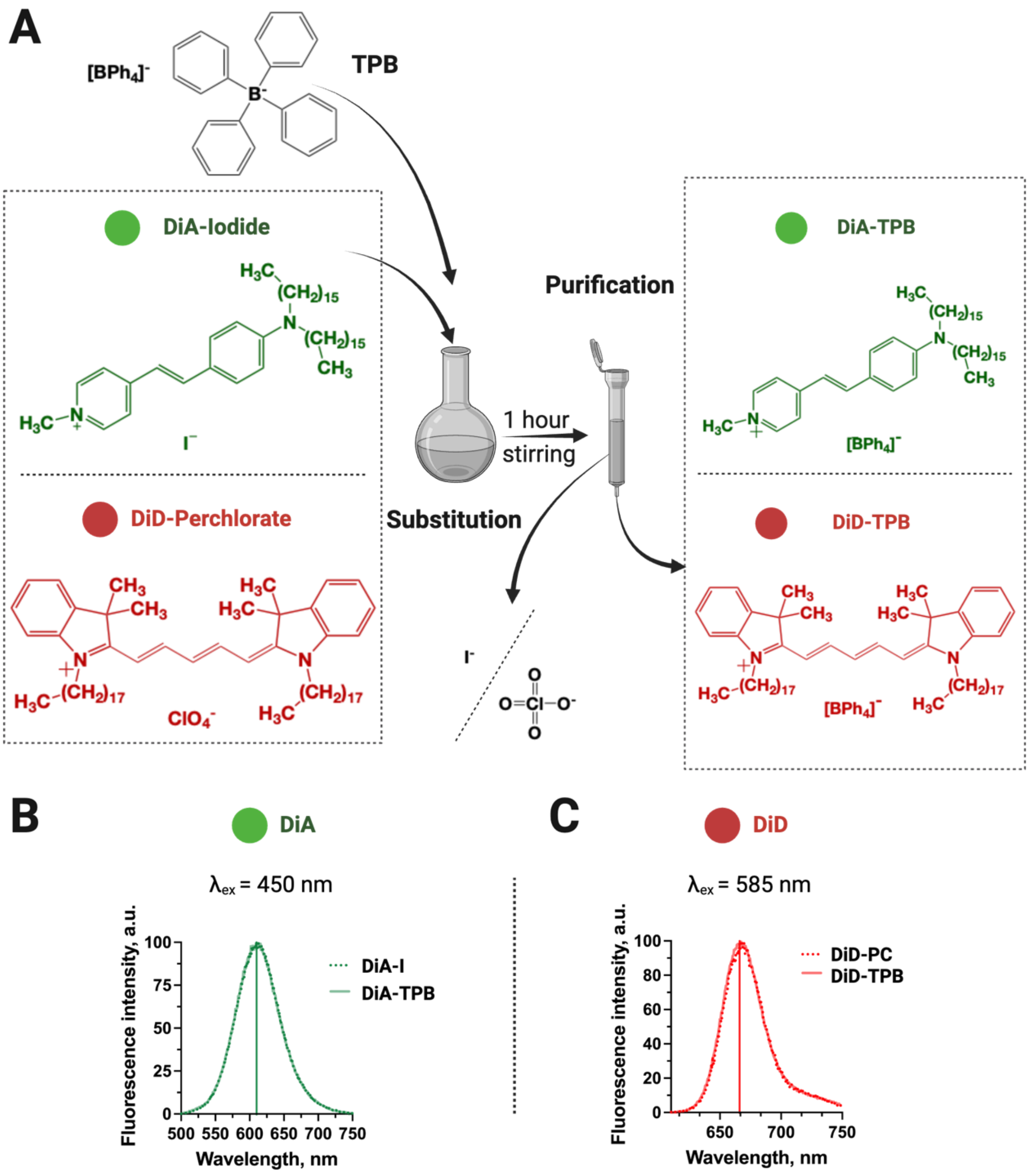

Figure S2. (A) Schematic representation of the counterion substitution protocol. Sodium tetraphenylborate (TPB) was added to the commercial fluorophores (DiA-iodide and DiD-perchlorate) dissolved in ethyl acetate, and, after 1 h of stirring, the solvent was removed by evaporation, and the residue was dispersed in a mixture of dichloromethane and methanol (95/5, v/v). The mixture was passed through a silica column in the case of DiA and a filter in the case of DiD to remove the sodium iodide or perchlorate and the excess of TPB. Normalized emission spectra of (B) DiA and (C) DiD fluorophores in ethanol before and after the counterion substitution.

Encapsulation efficiency and fluorophore loading

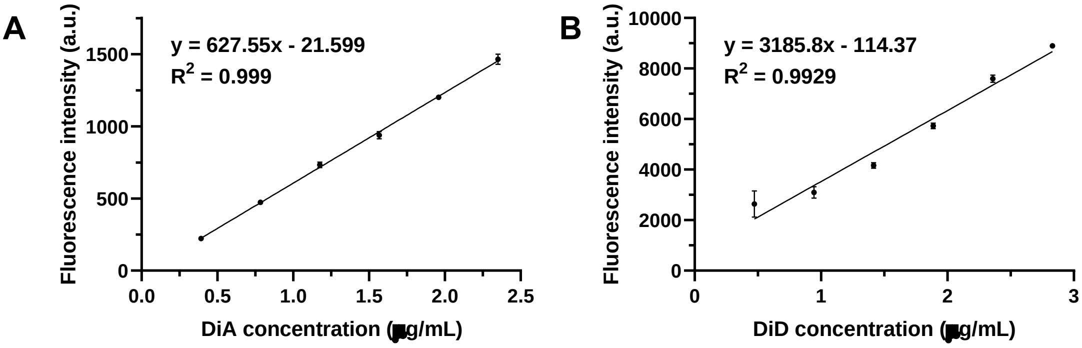

Figure S3. Calibration curve of (A) DiA and (B) DiD fluorophores for the determination of encapsulation efficiency and fluorophore loading

Release studies

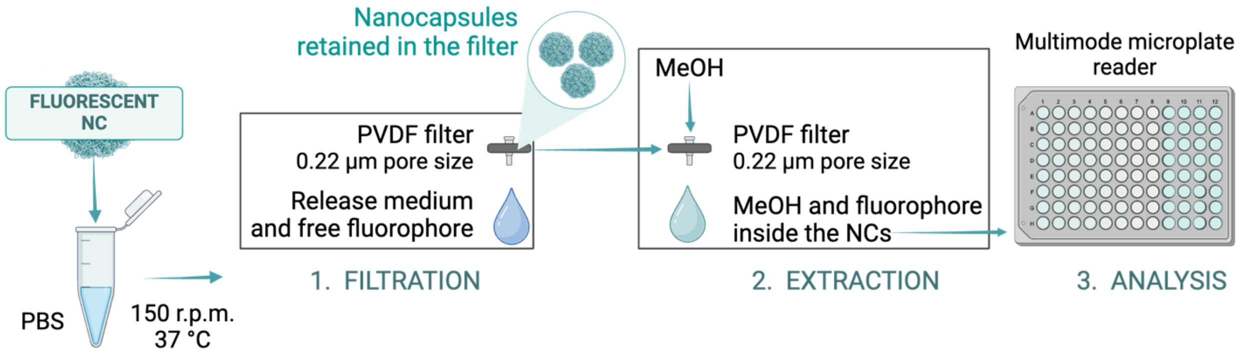

Scheme S1. Schematic representation of the release assay for the quantification of the fluorophore released by the NCs

Permeability/transcellular transport studies through Calu-3 model barrier

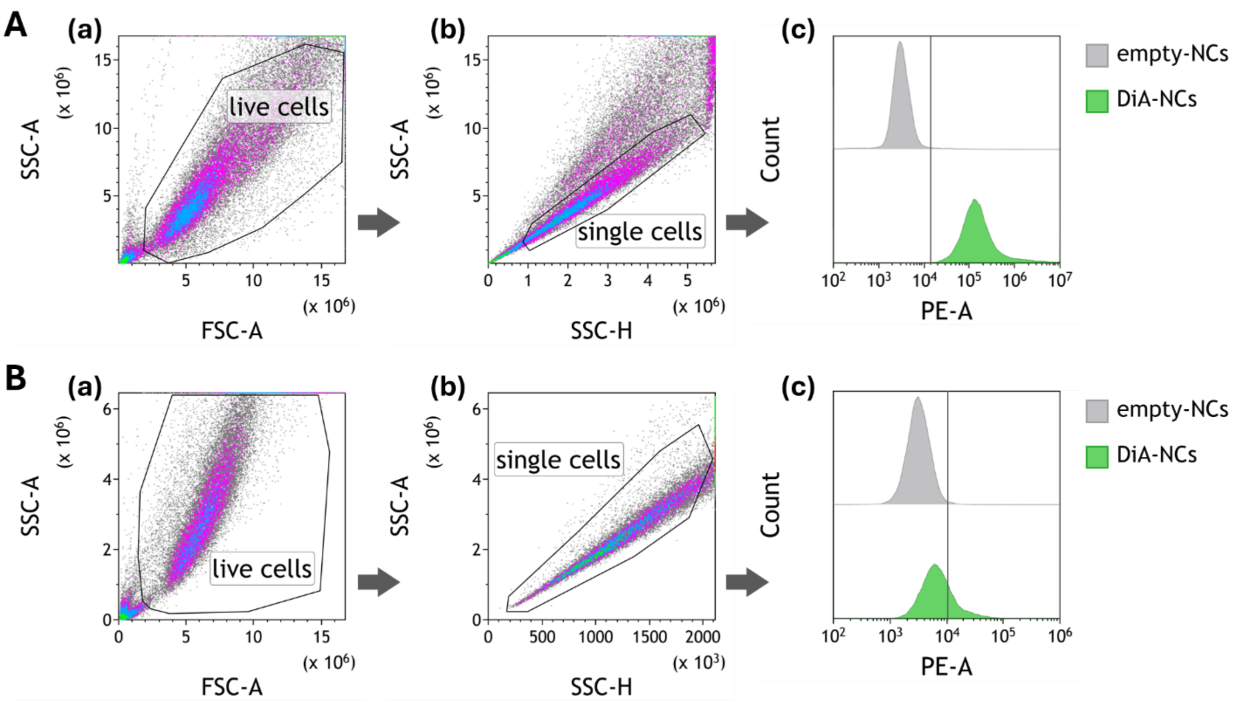

Figure S4. Flow cytometry gating strategy and gating hierarchy of a representative sample of (A) Calu-3 cells and (B) Balb-c 3T3 cells for analysis of nanocapsule cellular uptake. (a) Forward and side scatter gating (forward scatter area (FSC-A) vs side scatter-area (SSC-A)), identifying the cells of interest (cell-like events, live cells) based on their relative size and complexity and removing debris and other events that are not of interest. (b) Side scatter-height (SSC-H) vs SSC-A subgating to identify single cells. (c) Designating fluorescence negative and positive population gates using a histogram with non-fluorescent control (empty-NC treated) cells.

Preparation of the fluorophore-loaded NCs

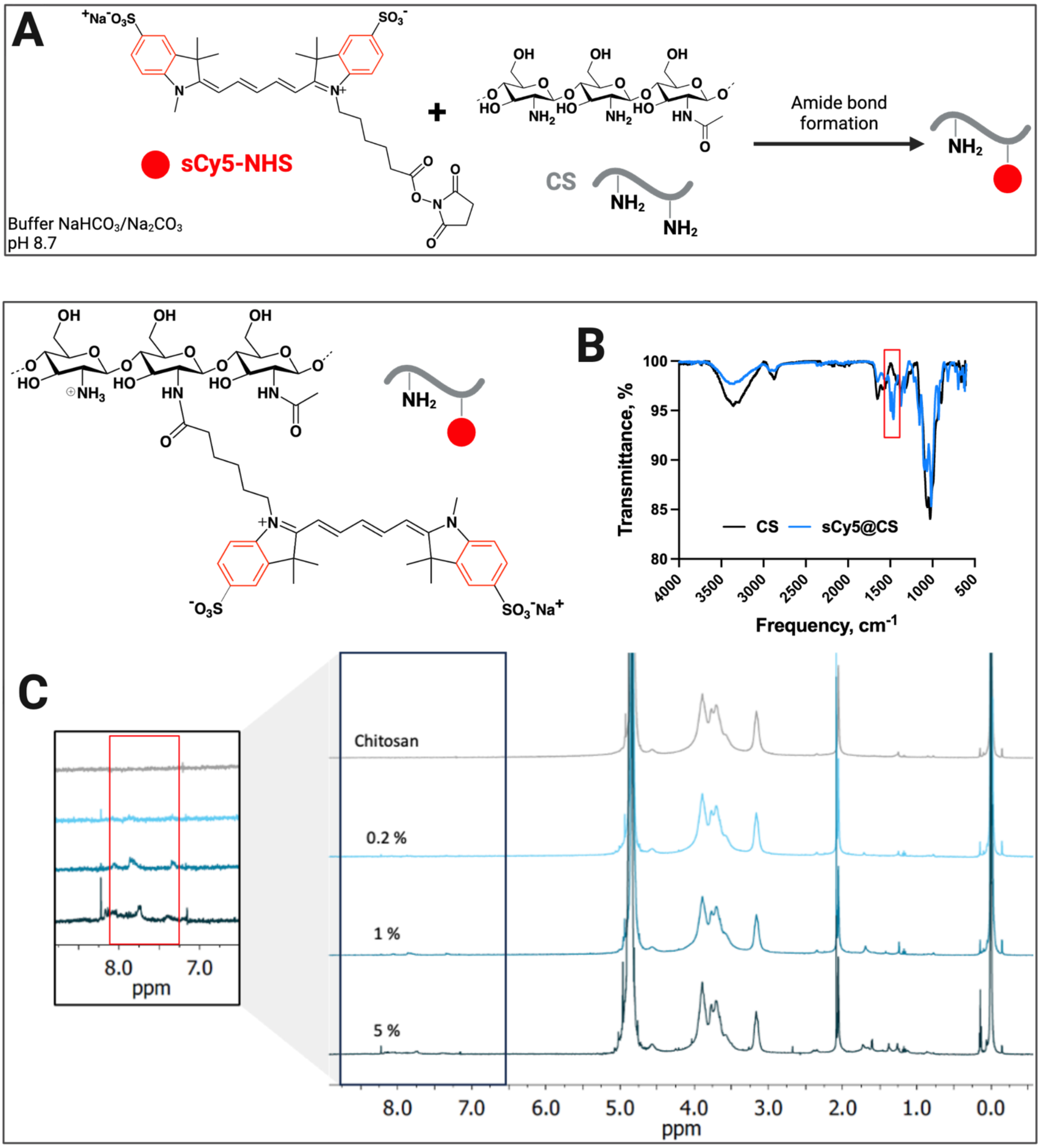

Figure S5. (A) Labelling of chitosan with sulfo-Cyanine 5-NHS ester (sCy5-NHS) for the formation of sulfo-Cyanine 5-chitosan. (B) FTIR spectrum of chitosan and chitosan functionalized with sCy5 (only the spectrum of sCy5@CS-5 is reported). (C) NMR spectra of chitosan and functionalized chitosan with different amount of sCy5. The red square indicates the presence of aromatic rings in the FTIR and NMR spectra.

Excitation and emission spectra of the fluorophores

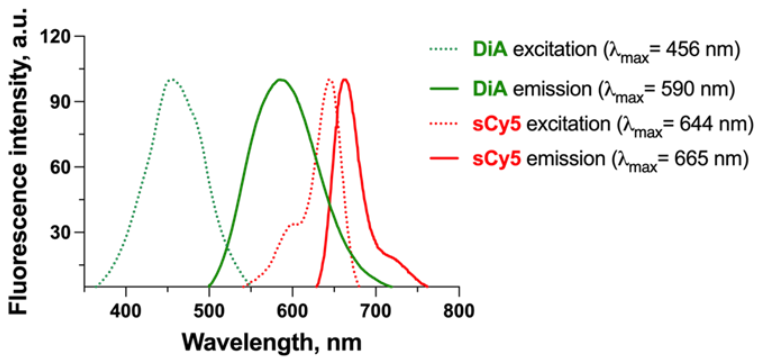

Figure S6. Excitation (dotted lines) and emission (plain lines) spectra of DiA (green) and sCy5 (red) fluorophores, as provided by the supplier.

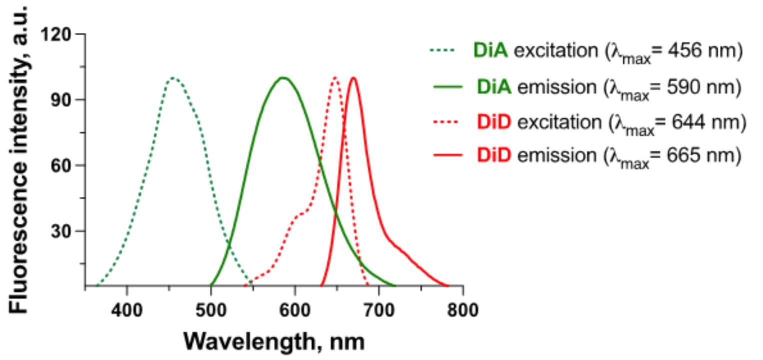

Figure S7. Excitation (dotted lines) and emissions (plain lines) spectra of DiA (green) and DiD (red) fluorophores, as provided by the supplier.

Encapsulation efficiency and fluorophore loading

Table S2. Encapsulation efficiency (EE) and fluorophore loading (FL) of nanocapsules (NCs) containing DiA, DiD, and FRET systems with different counterions. The data show a higher EE for DiA compared to DiD, with both exhibiting similar FL values, likely due to the comparable physicochemical properties of the two fluorophores, such as size and charge. Incorporating a more lipophilic counterion (TPB) resulted in a slight improvement in the EE and FL for DiA and the FL for DiD. The FRET-NCs also demonstrated improved EE for the fluorophores with TPB counterions. In contrast, sCy5-DiA-NCs exhibited a lower DiA loading compared to DiA- and FRET-NCs, potentially due to variations in CS substitution, indicating the role of the CS coating in stabilizing fluorophore loading.

Nanocapsule	EE, %	FL, nmol fluorophore/mgNC
DiA(I)-NC	82.9 ± 9.5	3.7 ± 0.4
DiA(TPB)-NC	93.6 ± 6.4	4.5 ± 0.3
DiD(PC)-NC	69.1 ± 3.2	3.4 ± 0.2
DiD(TPB)-NC	54.6 ± 1.6	4.4 ± 0.1
FRET(I-PC)-NC	DiA-I: 26.8 ± 5.7<br>DiD-PC: 37.1 ± 2.8	DiA-I: 4.7 ± 1.0<br>DiD-PC: 6.5 ± 0.5
FRET(TPB)-NC	DiA-TPB: 76.6 ± 4.7<br>DiD-TPB: 59.8 ± 6.0	DiA-TPB: 4.2 ± 0.3<br>DiD-TPB: 3.2 ± 0.3
sCy5@DiA(TPB)-NC	47.4 ± 0.4	2.4 ±0.0

Zeta potential, size, and morphology of the NCs

Table S3. ζ potential and hydrodynamic size of the nanocapsules

Nanocapsule	ζ potential, mV	hydrodynamic size mean, nm	PDI
Empty-NC	+21± 2	105 ± 22	0.3
DiA(I)-NC	+31 ± 2	98 ± 25	0.2
DiA(TPB)-NC	+33 ± 2	101 ± 8	0.2
DiD(PC)-NC	+32 ± 1	202 ± 37	0.2
DiD(TPB)-NC	+30 ± 1	91 ± 5	0.3
FRET(I-PC)-NC	+35 ± 2	75 ± 19	0.2
FRET(TPB)-NC	+34 ± 1	110 ± 21	0.4
sCy5@NC*	+13 ± 1	90 ± 10	0.4
sCy5@DiA(TPB)-NC*	+13 ± 1	87 ± 25	0.4

*NCs prepared using sCy5@CS-1.

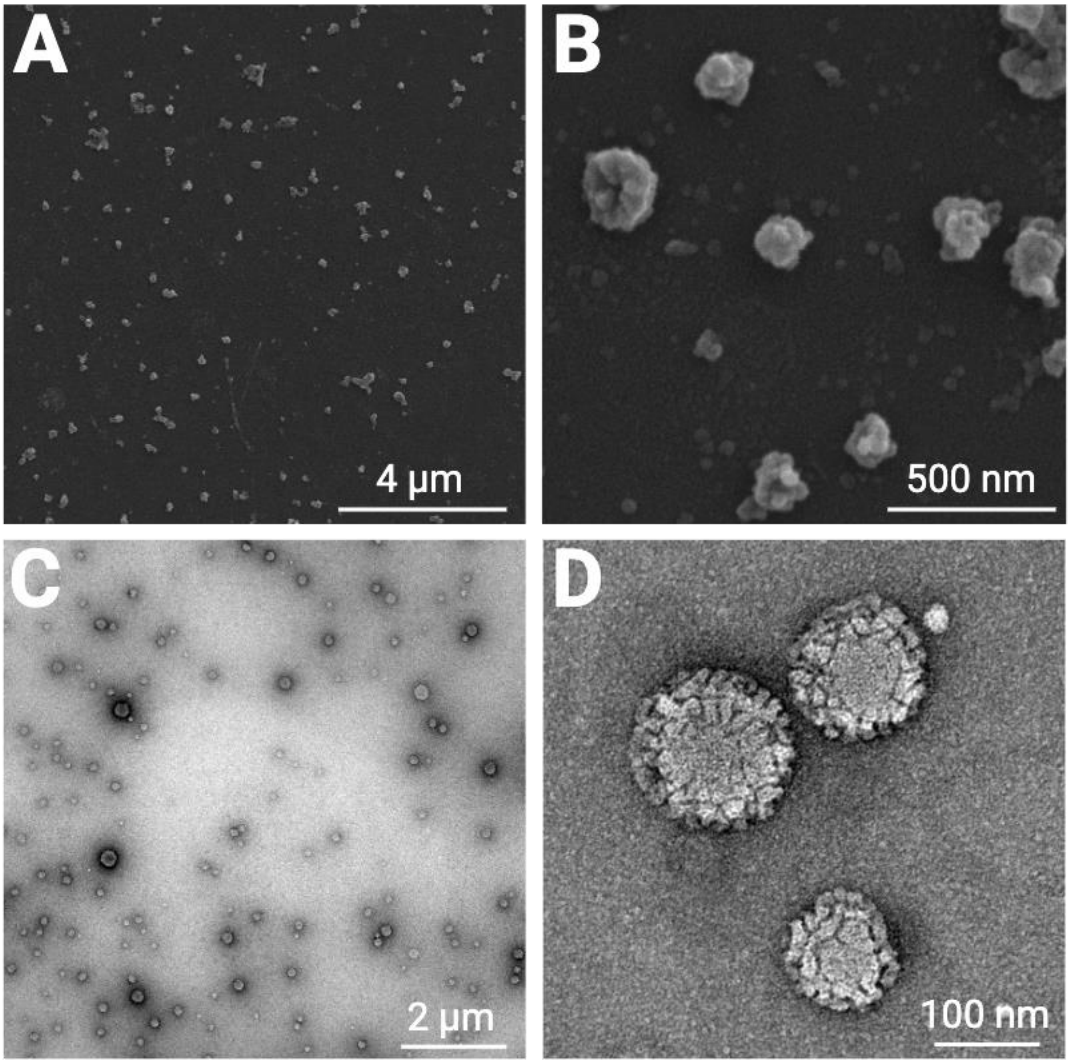

Figure S8. (A, B) SEM and (C, D) TEM micrographs of NCs with different magnifications

Emission spectra of sCy5@CS-NCs and sCy5@CS-DiA-NCs

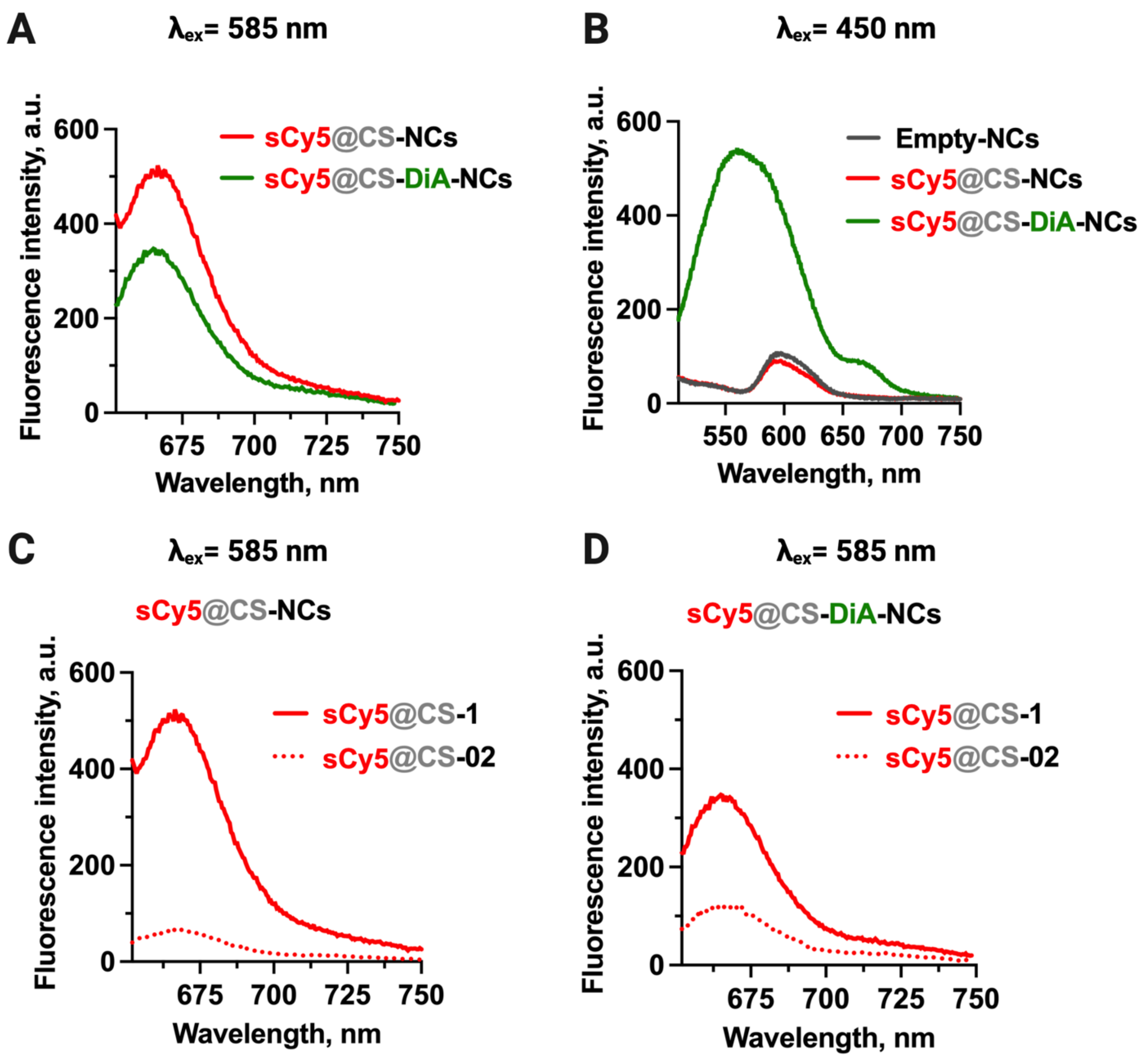

Figure S9. Emission spectra of sCy5@CS-NCs and sCy5@CS-DiA-NCs (with TPB counterion) at a nanocapsule concentration of 0.3 mg/mL, excited at (A) 585 nm and (B) 450 nm. Upon excitation at 585 nm, near the maximum emission wavelength of the DiA donor, we observed sCy5 (acceptor) emission at 671 nm, confirming the presence of sCy5 in both nanocapsules. When excited at 450 nm, no Förster Resonance Energy Transfer (FRET) was detected in sCy5@CS-DiA-NCs, indicating that the core and shell compartments are well-separated, with a distance greater than 10 nm between sCy5 in the shell and DiA in the core. This may be due to the formation of a surfactant layer between the compartments during synthesis. Fluorescent emission spectrum of (C) sCy5@CS-NC and (D) sCy5@CS-DiA-NC coated with sCy5@CS-02 (functionalized CS with 0.2% theoretical substitution degree, dashed line) and sCy5@CS-1 (functionalized CS with 1% theoretical substitution degree, solid line) (λ_{ex} 585 nm).

FRET signal evaluation by fluorimetry and flow cytometry

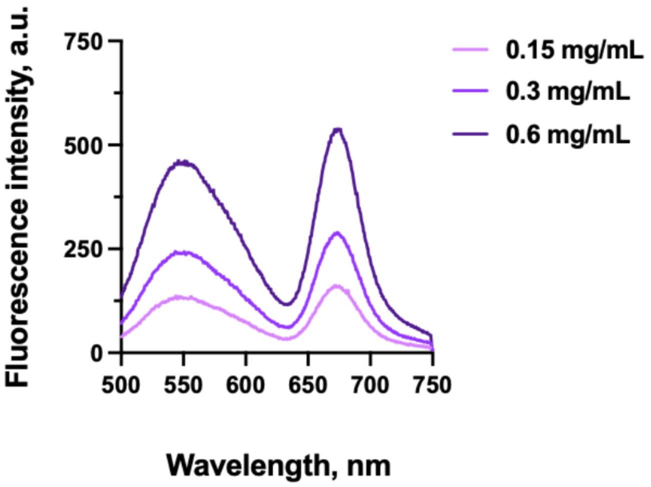

Figure S10. Fluorescence spectra of FRET-NCs at three different concentrations (0.15; 0.3; and 0.6 mg/mL). Excitation wavelength of 450 nm.

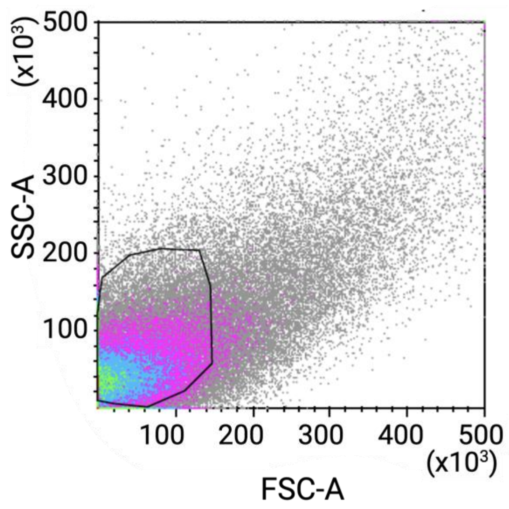

Figure S11. FSC-A vs SSC-A dot-plot of empty-NCs as an example.

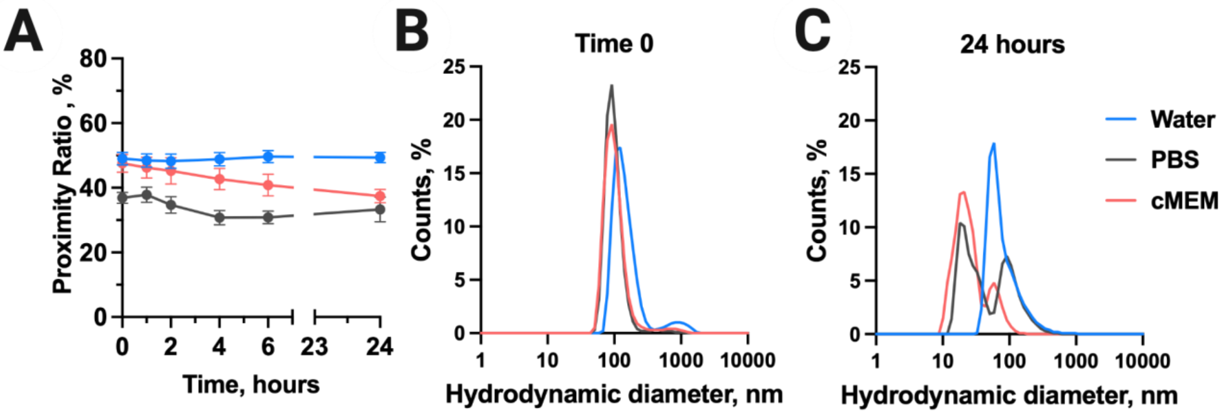

Figure S12. (A) The PR values obtained at different times in water, PBS, and cMEM with 1 mg/mL FRET-NC concentration. Experiments were carried out in duplicates; error bars represent standard deviation. Size distribution measured of FRET-NCs in water, PBS, and cMEM (B) at time 0 and (C) after 24 h of incubation.

NCs stability and NE diffusion through the CS shell in release assays

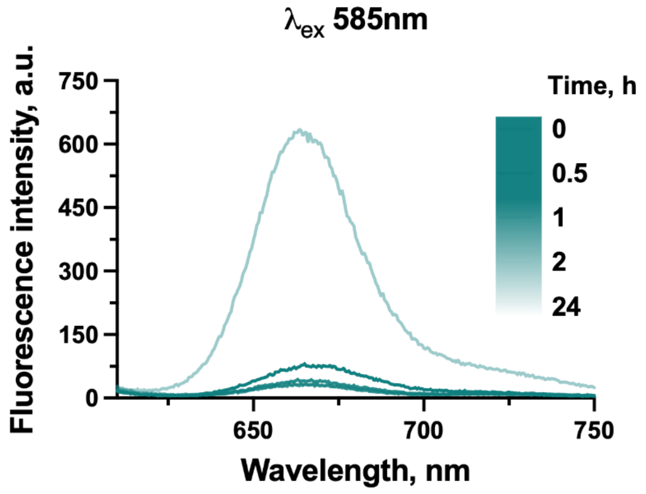

Figure S13. Fluorescence intensity spectra of the filtrates during the release assay in PBS at 37 °C with 1 mg/mL sCy5@CS-DiA-NC concentration at different time points (λex 585 nm). While negligible or no signal was detected between 0 and 2 hours, a great emission peak was collected after 24 hours of release in the filtrate. The appearance of the fluorescent signal of sCy5 can be related to the disruption of the CS shell and the release of the polymer in the medium.

***In vitro* cytotoxicity of the NCs**

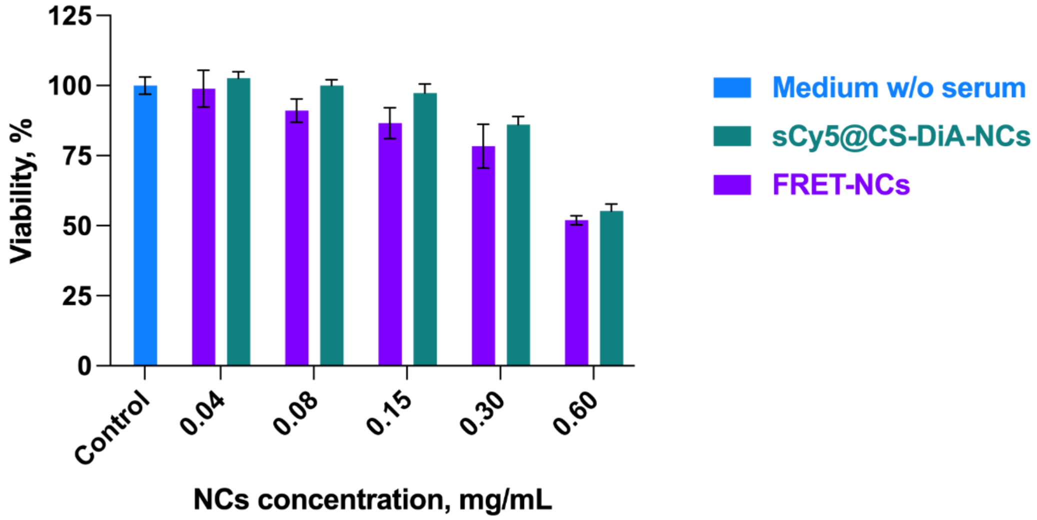

Figure S14. *In vitro* cytotoxicity of the nanocapsules. Cell viability of Calu-3 cells under different NC concentrations over 24 h. Experiments were carried out in duplicates; error bars represent standard deviation. The concentration of 0.3 mg/mL was considered the maximum concentration of NCs that could be added to the cells in further experiments without affecting cell viability markedly.

In vitro cellular uptake of the NCs

Cells were incubated with the NCs at different concentrations (0.075, 0.15, and 0.3 mg/mL), after washing and trypsinization, the intracellular fluorescence intensity of the cells was measured by flow cytometer, exciting them with a 488 nm laser. The fluorescence intensity of Calu-3 cells increases with increasing NC concentration in the case of DiA-NCs and FRET-NCs at the donor channel and at the acceptor channel in the case of FRET-NCs.

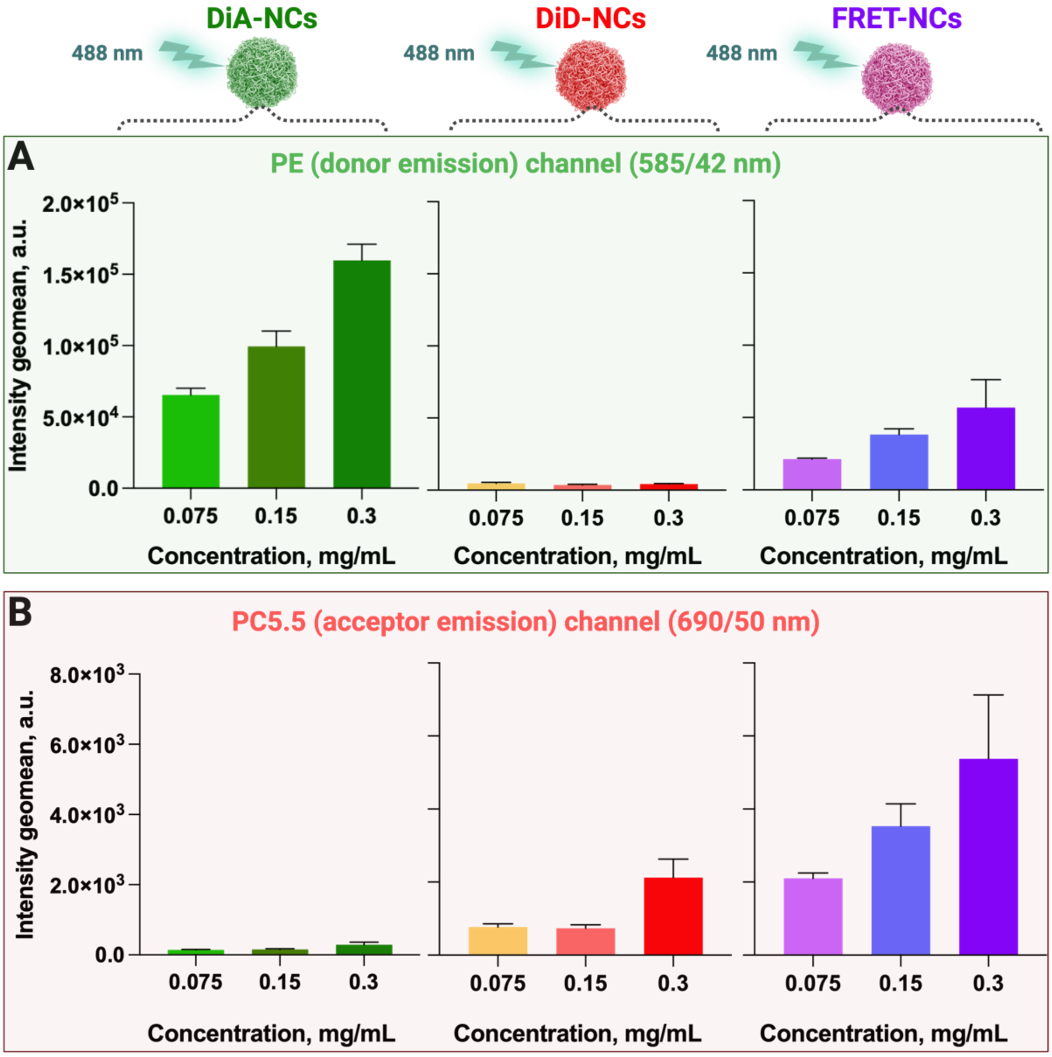

Figure S15. Mean intracellular fluorescence intensity of Calu-3 cells incubated for 24 h with DiA-, DiD-, and FRET-NCs at different concentrations determined by flow cytometry. Cells were excited using a 488 nm laser, fluorescent signal was collected at (A) the donor channel (B) the acceptor channel.

Establishment and characterization of a simplified *in vitro* nasal mucosal barrier model using Calu-3 cells

Calu-3 cells were cultured on Transwell® (TW) inserts with a PET membrane pore size of 3.0 μm to promote cell differentiation and the development of a polarized biological membrane.[1] The cells were seeded at a density of 4 x 10^5 cells/cm²,[2] and their monolayer formation was monitored using Trans-Epithelial Electrical Resistance (TEER), which assesses the electrical resistance across cell monolayers and indicates epithelial barrier integrity and tight junction (TJ) formation.[3] For Calu-3 cells on 3.0 μm pores, the minimum TEER value reported for a tight monolayer is 420 Ω cm².[4] Our cultures under liquid-liquid interface (LLI) conditions reached a maximal TEER value of approximately 450 Ω cm² after 10 days, indicating the formation of a robust epithelial barrier (Figure S16).

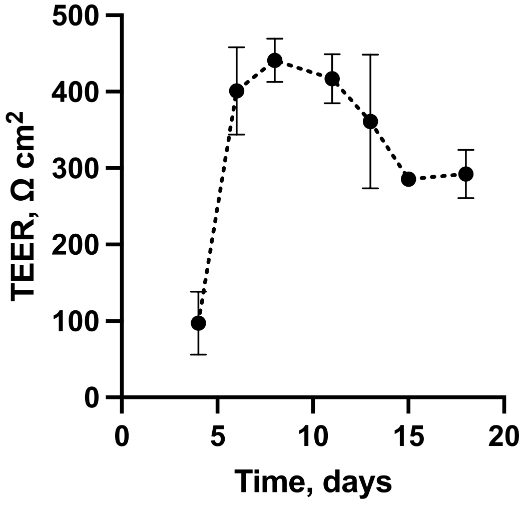

Figure S16. Trans-Epithelial Electrical Resistance (TEER) values of Calu-3 cells cultured on Transwell inserts under liquid-liquid interface (LLI) conditions

Structural and histological analyses were performed using SEM and TEM to further confirm the monolayer formation and functional integrity of the Calu-3 cell barrier. SEM images revealed that the cells were tightly packed, displaying regions of contact and a high density of microvilli, identified as tubular protrusions on the cell membrane (Figure S17A, B). Unlike Kreft *et al.*, who observed finger-shaped or protruding microvilli, we found microvilli appeared flattened in our samples, possibly due to differences in sample preparation.[2] Indeed, TEM imaging revealed well-defined microvilli about 500 nm in length, consistent with descriptions in liquid-liquid interface models (Figure S17C, D).[2] TEM also showed a developed endomembrane system with abundant rough endoplasmic reticulum (RE) and Golgi apparatus (GA), indicating active cellular processes. Additionally, tight junctions (TJ), desmosomes, and numerous secretory vesicles were observed, highlighting that Calu-3 cells differentiate into goblet cells under *in vitro* conditions.[5]

The differentiation of Calu-3 cells *in vitro* is characterized by the presence of mucus-secreting cells, reminiscent of the physiology of the bronchial epithelium *in vivo*. To verify mucus production, we used the alcian blue (AB) staining technique, which stains acidic mucins (sulfated and carboxylated acid mucopolysaccharides and sialomucins) blue due to the copper content in the dye. Figure S17E depicts a sagittal section of a TW insert with cells cultured under LLI conditions for three weeks. A monolayer of cells is visible on the TW membrane, appearing flattened rather than cuboidal, as seen in TEM images (Figure S17E, inset). This flattening may be due to differences in sample preparation; TEM samples are embedded in solid epoxy resin, while histological samples are embedded in softer paraffin, leading to distortion during cutting. Despite this structural distortion, the histological staining effectively demonstrated cellular differentiation. The AB staining highlighted the cytoplasm in pink and mucus components, such as acidic proteoglycans and glycoproteins, in blue, confirming the differentiation of Calu-3 cells into mucus-producing cells.

Another feature of the Calu-3 cellular monolayer is the presence of TJs. TJ formation in Calu-3 cells occurs when the cells reach a certain level of confluence or density.[1,2,6,7] To confirm the presence of TJs, immunostaining was used to stain Zonula-Occludens 1 (ZO-1), a key protein that assumes a crucial role in anchoring TJs and preserving their structural integrity, applying anti-ZO-1 antibody Alexa Fluor 488.[7] Immunofluorescence staining was performed both on cells seeded on glass slides (Figure S17F) and on cells cultured on TW (Figure S17G) and samples were observed through an inverted fluorescence microscope. Membranes appeared fluorescent after incubation with anti-ZO-1 antibody-AF488, specifically marking the locations where TJs were present (Figure S17F). A non-specific signal appearing on the membrane can be noticed in the images obtained when the cells were seeded on TW supports (Figure S17G). Since the TW membrane (without cells) did not show a similar signal after the incubation with the antibody, we attributed the noise to the difficult manipulation and the blending of the PET membrane on the glass slide, which presented challenges in obtaining optically distinct images, frequently resulting in images that lacked focus.

Overall, we successfully obtained a polarized cellular monolayer suitable for our permeability tests, which we characterized by combining TEER, SEM, and staining techniques. This simplified model was employed to carry out the validation of FRET-NCs.

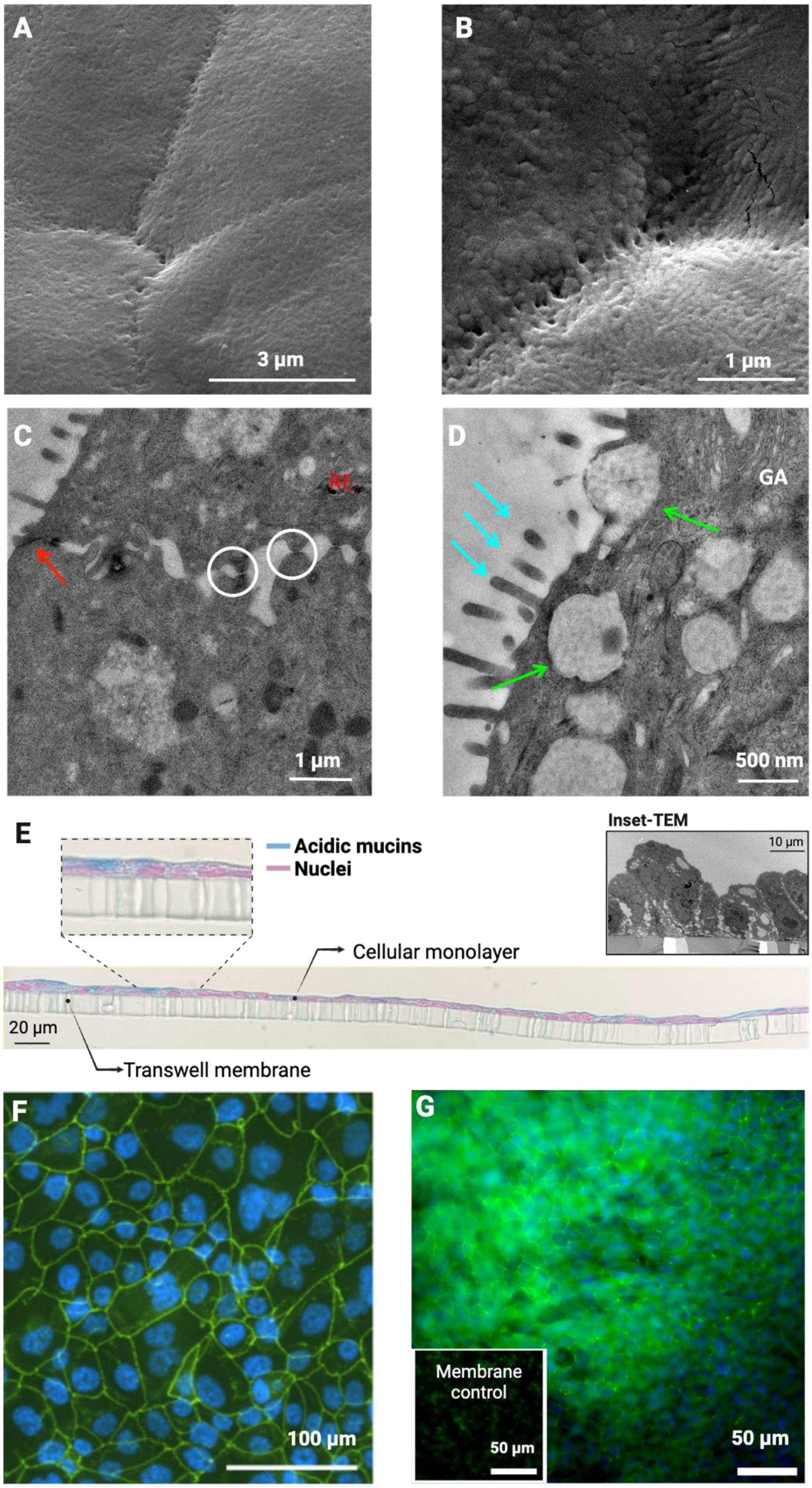

Figure S17. (A, B) SEM and (C, D) TEM micrographs of Calu-3 cells on TW under LLI conditions after 12 days. Cells exhibited TJs (red arrow), desmosomes (white circle), RE, microvilli (blue arrows), GA, and secretory vesicles (green arrow). (E) Alcian Blue staining of Calu-3 cells at three

weeks in culture on TW under LLI conditions. Acidic mucins are stained in blue, and nuclei are pink. Inset: TEM micrograph of the monolayer on TW. Immunolabeling of the anti-ZO-1 antibody (green) to ZO-1, expressed in TJs, in (F) cells cultured on a glass in a multi-well plate and (G) cells cultured during two weeks on a TW support at the LLI. Inset: bare membrane stained with anti-ZO-1. Nuclei in blue (DAPI).

NC transport across the Calu-3 barrier model

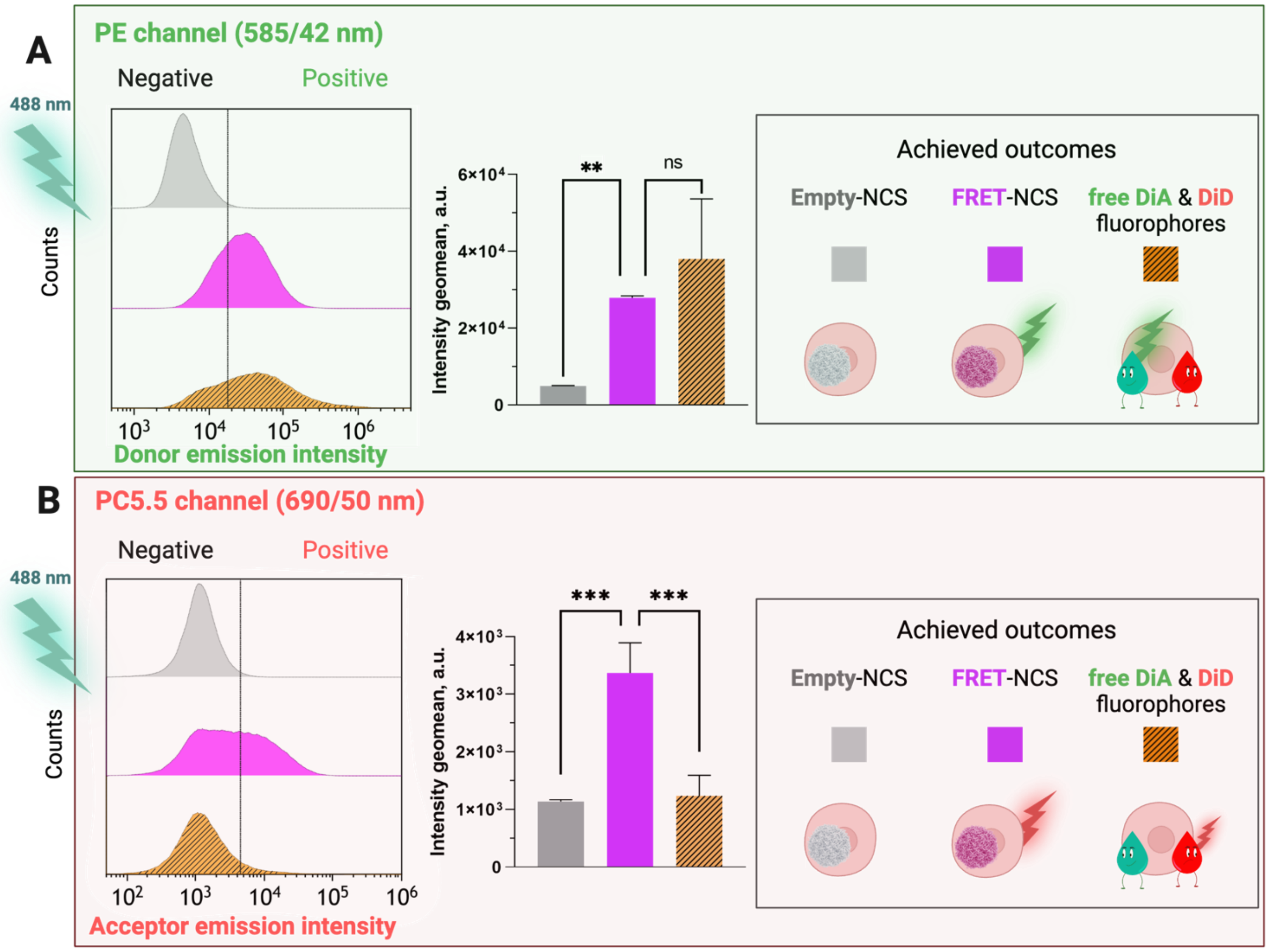

Figure S18. Flow cytometry histograms (left) and their corresponding geometric mean fluorescence intensity (middle) of Balb-c 3T3 target cells from the basal compartment of the Transwell system treated with empty-NCs, FRET-NCs and mixture of free DiA and DiD fluorophores (0.3 mg/mL NC concentration, loading equivalent fluorophore concentration, 24 h treatment). Cells were excited with a 488 nm laser and the emission was detected at the (A) PE channel (donor emission intensity, 585/42 nm) and at the (B) PC5.5 channel (acceptor emission intensity, 690/50 nm). One-way ANOVA, followed by Dunnet multiple comparison test. Data points are statistically different with $p \leq 0.005$ (**); and $p \leq 0.003$ (***), indicating significance.

References

(1) Silva, S.; Bicker, J.; Falcão, A.; Fortuna, A. Air-Liquid Interface (ALI) Impact on Different Respiratory Cell Cultures. *Eur. J. Pharm. Biopharm.* **2023**, *184*, 62–82. https://doi.org/10.1016/j.ejpb.2023.01.013.

(2) Kreft, M. E.; Jerman, U. D.; Lasič, E.; Hevir-Kene, N.; Rižner, T. L.; Peternel, L.; Kristan, K. The Characterization of the Human Cell Line Calu-3 under Different Culture Conditions and Its Use as an Optimized in Vitro Model to Investigate Bronchial Epithelial Function. *Eur. J. Pharm. Sci.* **2015**, *69*, 1–9. https://doi.org/10.1016/j.ejps.2014.12.017.

(3) Felix, K.; Tobias, S.; Jan, H.; Nicolas, S.; Michael, M. Measurements of Transepithelial Electrical Resistance (TEER) Are Affected by Junctional Length in Immature Epithelial Monolayers. *Histochem. Cell Biol.* **2021**, *156* (6), 609–616. https://doi.org/10.1007/s00418-021-02026-4.

(4) Agu, R. U.; Jorissen, M.; Willems, T.; Augustijns, P.; Kinget, R.; Verbeke, N. In-Vitro Nasal Drug Delivery Studies: Comparison of Derivatised, Fibrillar and Polymerised Collagen Matrix-Based Human Nasal Primary Culture Systems for Nasal Drug Delivery Studies. *J. Pharm. Pharmacol.* **2010**, *53* (11), 1447–1456. https://doi.org/10.1211/0022357011777981.

(5) Shen, B. Q.; Finkbeiner, W. E.; Wine, J. J.; Mrsny, R. J.; Widdicombe, J. H. Calu-3: A Human Airway Epithelial Cell Line That Shows CAMP-Dependent Cl- Secretion. *Am. J. Physiol. - Lung Cell. Mol. Physiol.* **1994**, *266* (5), 493–501. https://doi.org/10.1152/ajplung.1994.266.5.l493.

(6) George, I.; Vranic, S.; Boland, S.; Courtois, A.; Baeza-Squiban, A. Development of an in Vitro Model of Human Bronchial Epithelial Barrier to Study Nanoparticle Translocation. *Toxicol. Vitr.* **2015**, *29* (1), 51–58. https://doi.org/10.1016/j.tiv.2014.08.003.

(7) He, R. W.; Braakhuis, H. M.; Vandebriel, R. J.; Staal, Y. C. M.; Gremmer, E. R.; Fokkens, P. H. B.; Kemp, C.; Vermeulen, J.; Westerink, R. H. S.; Cassee, F. R. Optimization of an Air-Liquid Interface in Vitro Cell Co-Culture Model to Estimate the Hazard of Aerosol Exposures. *J. Aerosol Sci.* **2021**, *153*, 105703. https://doi.org/10.1016/j.jaerosci.2020.105703.
